# An Integrated Smart Sensor Dressing for Real-Time Wound Microenvironment Monitoring and Promoting Angiogenesis and Wound Healing

**DOI:** 10.3389/fcell.2021.701525

**Published:** 2021-08-06

**Authors:** Yuheng Zhang, Tian Li, Congying Zhao, Jinqing Li, Rong Huang, Qianru Zhang, Yongqian Li, Xueyong Li

**Affiliations:** ^1^Department of Burn and Plastic Surgery, Tangdu Hospital, Fourth Military Medical University, Xi’an, China; ^2^Air Force Hospital of Western Theater Command, Chengdu, China; ^3^School of Basic Medicine, Fourth Military Medical University, Xi’an, China; ^4^School of Software Center for High Performance Computing, Northwestern Polytechnical University, Xi’an, China; ^5^Key Laboratory of Micro/Nano Systems for Aerospace, Ministry of Education, Northwestern Polytechnical University, Xi’an, China

**Keywords:** wound monitoring, angiogenesis, smart dressing, wireless sensor, hydrogel (GelMA)

## Abstract

Prolonged chronic wound healing not only places great stress on patients but also increase the health care burden. Fortunately, the emergence of tissue-engineered dressings has provided a potential solution for these patients. Recently, the relationship between the wound microenvironment and wound healing has been gradually clarified. Therefore, the state of wounds can be roughly ascertained by monitoring the microenvironment in real time. Here, we designed a three-layer integrated smart dressing, including a biomimetic nanofibre membrane, microenvironment sensor and β-cyclodextrin-containing gelatine methacryloyl (GelMA + β-cd) UV-crosslinked hydrogel. The hydrogel helped increase the expression of vascular endothelial growth factor (VEGF) through hypoxia-inducible factor-1α (HIF-1α) to promote neovascularization and wound healing. The microenvironment sensor, combined with the biological dressings, exhibited satisfactory measurement accuracy, stability, durability and biocompatibility. A BLE4.0 antenna was used to receive, display and upload wound microenvironment data in real time. Such integrated smart dressings can not only achieve biological functions but also monitor changes in the wound microenvironment in real time. These dressings can overcome the challenge of not knowing the state of the wound during the healing process and provide support for clinical work.

## Introduction

A poor local blood supply is a common feature of chronic wounds, which can have various causes (Dinh and Veves, 2005; Chao and Cheing, 2009; Cahn et al., 2016). Therefore, determining how to promote wound angiogenesis to accelerate healing is a very important and challenging issue. Angiogenesis in wounds is a complex and multistep process that mainly occurs in the proliferation stage of healing, during which vascular endothelial cells (VECs) proliferate and form a neovascularization network through dynamic changes in shape and behavior. With the rapid development of material science and stem cell tissue engineering, active biological dressings have shown great potential in wound treatment (Yang et al., 2014; Nurhasni et al., 2015; Peh et al., 2015; Shi et al., 2015). Biomedical hydrogels are one example of such dressings. Compared with traditional dressings, biomedical hydrogels have obvious advantages in terms of, for example, antibacterial properties and biocompatibility (Shevchenko et al., 2010). For example, biomedical hydrogels can better maintain the moist environment of the wound, and their more similar structure to that of the extracellular matrix (ECM) can provide scaffolds for cell adhesion and migration. The three-dimensional (3D) culture of cells based on a hydrogel matrix can better simulate the real ECM environment *in vivo*. Gelatine methacryloyl (GelMA) has high potential as a tissue engineering material because of its good biological properties and controllable physical properties, and GelMA hydrogels produced by UV crosslinking are widely used in the field of regenerative medicine (Yue et al., 2015). The formation of vascular lumens by endothelial cells is a critical step in the angiogenic process that occurs during invasion and growth of the incipient vascular sprout (Iruela-Arispe and Davis, 2009). Some researchers have used 3D printing technology to carve a lumen structure in GelMA hydrogels and found that these hydrogels could accelerate the proliferation and migration of VECs and promote wound neovascularization (Lee and Mooney, 2001; Aubin et al., 2010; Nichol et al., 2010; Qi et al., 2010). Angiogenesis is a very complex process involving many factors. Vascular extracelluar matrix also plays an important role in the development of the functional circulation (Davis et al., 2011). As a pharmaceutical excipient, β-cyclodextrin (β-cd) is widely used to increase cell permeability, thus increasing the uptake of small molecules such as glucose and nanoparticles. Recently, it has been found that β-cd can also adjust the physical and chemical properties of hydrogels and improve their biocompatibility (Niu et al., 2018; Bezerra et al., 2020).

Recently, the relationship between the wound microenvironment and healing state has been gradually elucidated through in-depth research (Junker et al., 2013). The local temperature, a common indicator, has received more attention (Kruse et al., 2015). However, there are few suitable methods to monitor the local temperature in real time during clinical work. The traditional mercury thermometer cannot be used to easily measure the wound temperature, as it does not conform to the wound and takes a long time to equilibrate. Thus, this equipment is obviously not suitable for clinical application. Due to the many layers of dressings, an infrared thermometer is also not suitable. With the intelligence and miniaturization of sensors, mature and commercialized microsensors have gradually emerged. Smart dressings can enable real-time monitoring of the wound microenvironment combined with flexible substrate-integrated circuit and wireless communication technology, providing medical staff with wound data with which to assess the healing state (Payam et al., 2017). Matzeu et al. (2016) developed a wireless sensor for monitoring skin temperature, which mainly consists of a thermistor and radio-frequency identification (RFID) technology and has been applied in dressings and bandages. Pang et al. (2020) designed a double-layer smart flexible electronics-integrated wound dressing by combining flexible electronic components with an antibacterial hydrogel, which enabled the wound temperature to be monitored in real time and allowed antibiotics to be released from the hydrogel by *in situ* ultraviolet (UV) irradiation. This series of studies demonstrates that it is feasible to monitor the wound microenvironment using sensors.

Herein, we report an integrated smart dressing that combines a microenvironment sensor, a biomimetic nanofibre membrane and a GelMA + β-cd UV-crosslinked hydrogel. The dressing could not only be used to monitor the wound microenvironment in real time but could also promote wound angiogenesis and healing. The wound microenvironment was continuously monitored by the integrated sensor chip, and the data were transmitted to a portable terminal device (e.g., smartphone) via a Bluetooth low-energy (BLE) 4.0 antennae and displayed on a customized app. The integrated smart dressing both retained the diversified functions of biological dressings and achieved real-time monitoring of the wound microenvironment. This application overcomes the challenge of not knowing the state of the wound during the treatment process and provides support for clinical work.

## Materials and Methods

### Fabrication of the Biomimetic Nanofibre Membrane

The biomimetic nanofibre membrane used in this study was a nanofibrous matrix assembled in a layer-by-layer (LBL) manner by chitosan/collagen, which we designed and manufactured in a previous study. This membrane enhanced cell migration and further promoted skin regeneration by upregulating the secretion of ECM proteins and triggering the integrin/FAK signaling pathway. The production method has been described in detail in our previous research (Huang et al., 2014, 2015, 2019; Cui et al., 2017).

### Fabrication of the Microenvironment Sensor

The sensor consisted of a sensing chip and a control module, which were encapsulated in white plastic cases. The sensing chip was a printed circuit board with a welded temperature and humidity sensor (HDC1080DMBR, Texas Instruments, United States) and air-pressure sensor (LPS22HBTR, STMicroelectronics, Switzerland). The control module included a BLE antenna (RAINSUN, Taiwan), digital controller (NRF51822, NORDIC, Norway) and power management device (TP4056, NanJing Top Power ASIC Corp., China). The sensing chip and control module were connected by wires and joints and controlled by a switch; we have used these devices in previous research to predict wound infection (Zhang et al., 2021).

### Preparation and Characterization of Hydrogels

#### Synthesis of Hydrogels

To generate hydrogels, freeze-dried GelMA prepolymer was completely dissolved in DPBS at a concentration of 5% (w/v). After full dissolution, β-cd was added to the solution to a final concentration of 0, 2, 3 and 4% (w/v) in a 60°C water bath. When the solution was clarified, 0.25% (w/v) 2-hydroxy-4′-(2-hydroxyethoxy)-2-methylpropiophenone was added as a photoinitiator, and the prepolymer solution was photocrosslinked through UV light irradiation (SPECTROLINKER XL-1500 UV CROSSLINKER, United States) for 15 min at room temperature (RT) to form hydrogels. The pH of the prepolymer solutions and hydrogels was measured using a SevenCompact pH meter (S210, METTLER TOLEDO INSTRUMENT CO., LTD., China) before and after crosslinking.

#### Characterization

##### Transparency

To determine the formability and transparency of the prepared GelMA + β-cd gradient composite hydrogel discs, images were of various post-gelation samples placed on a graduated steel ruler were captured. In addition, a UV-2800 UV-VIS spectrophotometer (UNICO INSTRUMENT CO., LTD., China) was used to detect the transmittance (a.u.) of the prepared samples at wavelengths ranging from 450 to 925 nm. Deionized (DI) water was applied as a control.

##### Surface and internal morphology

The surface and cross-sectional morphologies of the GelMA prepolymer and lyophilized crosslinked hydrogels were examined by scanning electron microscopy (SEM). Hydrogels were cut into thin, circular slices with a diameter of approximately 5 mm and lyophilized in a lyophilizer (LGJ-18C, China) for 12 h. Then, the samples were mounted onto an aluminum stage and coated with gold in a sputter coater (Hitachi, Tokyo, Japan). The surface and cross-sectional morphologies were examined by SEM at 10 mA with an accelerating voltage of 10 kV. The surface roughness of different β-cd gradient composite hydrogels was determined using a Nanoscope IV atomic force microscopy (AFM) system (Innova, Bruker AXS., United States) in tapping mode and is expressed as height and using phase images. Three randomly selected areas of the surface with a size of 10 × 10 mm (x, y direction) were scanned. To describe the topography and roughness of the substrates, the roughness parameter for the surface [i.e., the root mean square roughness (Rq)], was calculated by Nanoscope analysis. In addition, the porosity of the prepared gels (1 cm × 1 cm) was determined using an AutoPore IV9500 automatic mercury porosimeter (Micromeritics, United States) at 3000 psi.

##### Fourier transform infrared (FT-IR) spectroscopy and X-ray diffraction (XRD) analysis

To confirm the successful reaction of MA with gelatine and grafting of β-cd onto GelMA, the chemical structures of the GelMA prepolymer, β-cd, and UV-crosslinked GelMA + β-cd gradient composite hydrogels were analyzed based on their infrared absorption bands detected with an FT-IR spectrometer (PerkinElmer Spectrum 2000, Waltham, MA, United States). In addition, the lyophilized hydrogel samples were ground with KBr powder, and the mixture was pelletized at RT. The spectra were recorded in the wavenumber range of 400-4000 cm^–1^ with 64 scans per sample cycle and a resolution of 4 cm^–1^. The composition of the as-prepared samples was analyzed using energy-dispersive X-ray (EDX) spectroscopy. XRD was performed using a type D/max-Ra diffractometer (Rigaku Co., Japan) with a Cu target and Kα radiation (λ = 0.154 nm) at 40 kV and 50 mA at RT. The scanning rate was 0.5°/min, and the scanning scope of 2θ was 1–10° and 5–60° in fixed time mode.

### Cytotoxicity Assay

One milliliter of prepolymer solution was crosslinked into a gel by UV light, soaked in 75% ethanol, sterilized overnight, fully washed and swollen with aseptic PBS. The gel was soaked in 20 mL of endothelial cell medium and incubated at 37°C for 48 h. After the incubation period, the so-called extracts were obtained and filtered through a 0.22-mm filter prior to the following experiments. The experimental protocols for the use of human dermal microvascular endothelial cells (HDMECs) were approved by the Ethics Committee for Animal Experimentation of the Fourth Military Medical University (No. 201903-58). HDMECs (Procell, China) were maintained in endothelial cell medium (ScienCell, United States) supplemented with 10% fetal bovine serum, 100 units per mL penicillin/streptomycin, 30 μg/mL endothelial cell growth supplement and 25 U/mL heparin sodium. The cells were maintained in a humidified atmosphere of 95% air and 5% CO2 at 37°C. The culture medium was replaced every 2 days until the cells were confluent.

#### Cell Counting Kit 8 (CCK-8) Assay

The cytotoxicity of the extracts to HDMECs was measured by CCK-8 assay. A total of 1 × 10^4^ HDMECs were seeded in 96-well microtiter plates, incubated in 200 μL of endothelial cell medium and cultured with the extracts for 1, 2, 3, 5, 7, and 10 days. Cells cultured in endothelial cell medium were used as a control. At each time interval, CCK-8 solution (7seabiotech, China) was added to the culture system and incubated for 3 h at 37°C. Subsequently, the cell viability was evaluated by determining the absorbance at 495 nm using a microplate reader (Model 200 PRO, TECAN, United States). All the experiments were repeated thrice.

#### Flow Cytometry Analysis

Apoptotic HDMECs were differentiated from viable and necrotic HDMECs by double staining with fluorescein isothiocyanate (FITC)-conjugated annexin V and propidium iodide (PI) (BD Pharmingen, CA, United States). HDMECs (1 × 10^6^/mL) were implanted on GelMA hydrogels containing different amounts of β-cd, incubated in 6 mL of endothelial cell medium for 3 days, harvested, washed, and double stained with an Annexin V-FITC apoptosis detection kit according to the manufacturer’s instructions. Samples were incubated at RT for 15 min in the dark with Annexin V and PI and quantitatively analyzed by a FACSCalibur flow cytometer (Beckon Dickinson, Mountain view, CA, United States).

### Human Dermal Microvascular Endothelial Cells Proliferation and Lumen Formation on Hydrogels

The 2% β-cd hydrogel was crosslinked *in situ* in a confocal dish and then sterilized with 75% alcohol for 48 h. Then, the hydrogels were soaked in PBS (pH 7.4) overnight to remove residual crosslinking solution and were equilibrated in culture medium for 24 h before cell culture. Primary HDMECs were initially seeded on the hydrogels at a density of 8000 cells/cm^2^, with endothelial cell medium supplied under normal conditions. Cells cultured in a blank confocal dish were used as controls. After 5 days of culture, 4% paraformaldehyde was added to fix the cells when the cells fused to form a large number of lumens for follow-up experiments.

### Hydrogel Degradation and Promotion of Angiogenesis *in vivo*

The experimental protocol was approved by the Ethics Committee for Animal Experimentation of the Fourth Military Medical University (No. 201903-58) and was in accordance with the Guide for the Care and Use of Laboratory Animals of the National Institutes of Health. The *in vivo* degradation of the GelMA + β-cd hydrogels was evaluated by subcutaneous implantation in mice. A total of 24 male BALB/c mice (20 ± 5 g) were used in this study. All surgeries were performed under anesthesia. After anaesthetization with 1% pentobarbital sodium for 5 min, the hair on the back of each mouse was shaved, the skin on their backs was dissected over approximately 20 mm, and the outer membrane under the skin was stripped. The cells were labeled with PKH26 and loaded on the hydrogel (with 2% β-cd), which was implanted after dissection and sutured under aseptic conditions. Mice that underwent the same operation but did not undergo hydrogels implantation were used as the control. After surgery, the animals were given antibiotics twice daily for 48 h. The mice were sacrificed to analyze the size of residual hydrogel at 3, 7, 14, and 21 days after implantation, and samples of the implant site were collected for analysis on the 3rd and 7th days. Three mice were used for each data point. The harvested tissue blocks containing the hydrogel were washed in PBS and frozen in liquid nitrogen.

### Assembly of Integrated Smart Dressing

The integrated smart dressing was assembled following these steps. First of all, immerse the oxirane sterilized sensor chip in the prepolymer solution which had been filtered through a 0.22-mm filter, and then UV-crosslink *in situ*. Or sculpt shapes in the prepared hydrogel, embedding the sterilized sensor chip. After that, cover the sterilized biomimetic nanofibre membrane on the side that would contact with the wound.

### Characteristic Detection of Integrated Smart Dressing

#### Accuracy and Stability Tests

The sensor testing method was carried out as follows. Briefly, the sensor chip was placed in a constant-temperature shaker (LYZ-100B, LONGYUE, China), and the temperature was gradually increased. One hundred pairs of data were obtained at the same time in the range of 35 ∼ 42°C (possible temperature range of wounds), and 30 pairs of data were randomly selected for analysis. In addition, the stability was tested by placing the sensor chip in an incubator (Thermo Fisher Forma 371, Thermo, United States) with temperature settings of 33, 35, 37, 39, and 41°C for 15 min, separately, and the data were recorded every 30 s. All the experiments were repeated three times.

#### Durability and Cytotoxicity Tests

To simulate moist and liquid wound environments *in vivo* at 37°C, the sensor chip was immersed in endothelial cell medium for 3, 5, and 7 days and buffer at pH 4, 7, and 10 for 3 days. At each time point, the sensor chip was directly powered, and 30 sets of temperature data were read. The smart dressing immersed in Dulbecco’s modified Eagle medium (DMEM) at 37°C for 24 h, followed by extract solution collection. The cytotoxicity of the extract to L-929 cells was measured by CCK-8 assay. A total of 1 × 10^4^ L-929 cells were seeded in 96-well microtiter plates, incubated in 200 μL of DMEM and cultured with the extract solution for 1, 3, 5, and 7 days. Cells cultured in DMEM were used as a control. At each time interval, CCK-8 solution (7seabiotech, China) was added to the culture system and incubated for 3 h at 37°C. Subsequently, the cell viability was evaluated by determining the absorbance at 495 nm using a microplate reader (Model 200 PRO, TECAN, United States). All the experiments were repeated three times.

### Monitoring and Promotion of Wound Healing by Integrated Smart Dressings

The animal experiments were performed in accordance with protocols approved by the Ethics Committee of Tangdu Hospital (No. 201903-58) and in accordance with the NIH Guide for the Care and Use of Laboratory Animals or other appropriate guidelines. New Zealand rabbits (Experimental Animal Center of Fourth Military Medical University) were allowed to acclimate for 2 weeks before the experiments. A total of 5 animals were used for this study. All animals were anesthetized by intravenous injection with pentobarbital sodium (30 mg/kg) before the surgical procedure. The area was disinfected with povidone-iodine solution and then removed with 75% ethanol after the hair was shaved. Two standardized 3.5-cm-diameter, full-thickness wounds were created between the crest of the shoulders and the coccygeal tuberosity on either side of the back at 1.5 cm on each side of the median line of the dorsal spine. The wounds were fixed with plastic rings to prevent contraction. Each wound area created was approximately 9.6 cm^2^, and the total wound area was less than 10% of the animal’s total body surface area. All procedures were performed by the same surgeon. Left back wounds were bound with smart dressings and fixed with transparent film, and wounds on the other side were routinely bandaged with gauze as a control. The wound temperature was continuously monitored after modeling, and the values were recorded at the same time point. To eliminate the effect of anesthesia on the wound temperature of animals, the wound temperature at 12 h after the operation was recorded as the value on the 0th day. On the 0th, 3rd, 7th, and 14th days after the operation, the wound area was imaged by a digital camera, and samples were collected on the 3rd and 7th days. The obtained wound specimens in each group were fixed in 4% paraformaldehyde at 4°C overnight, dehydrated with a graded series of ethanol, embedded in paraffin and sectioned.

### Statistical Analysis

Each experiment was repeated at least 3 times on different days, and the quantitative results are presented as the mean ± SD. The unpaired Student’s *t*-test, paired-samples *t*-test and one-way ANOVA were utilized to evaluate the significance among control and experimental samples. ^∗^*P* < 0.05 was considered statistically significant, and ^∗∗^*P* < 0.01 and ^∗∗∗^*P* < 0.001 were considered extremely significant.

## Results

### Structure of the Integrated Smart Dressing

**FIGURE 1:**
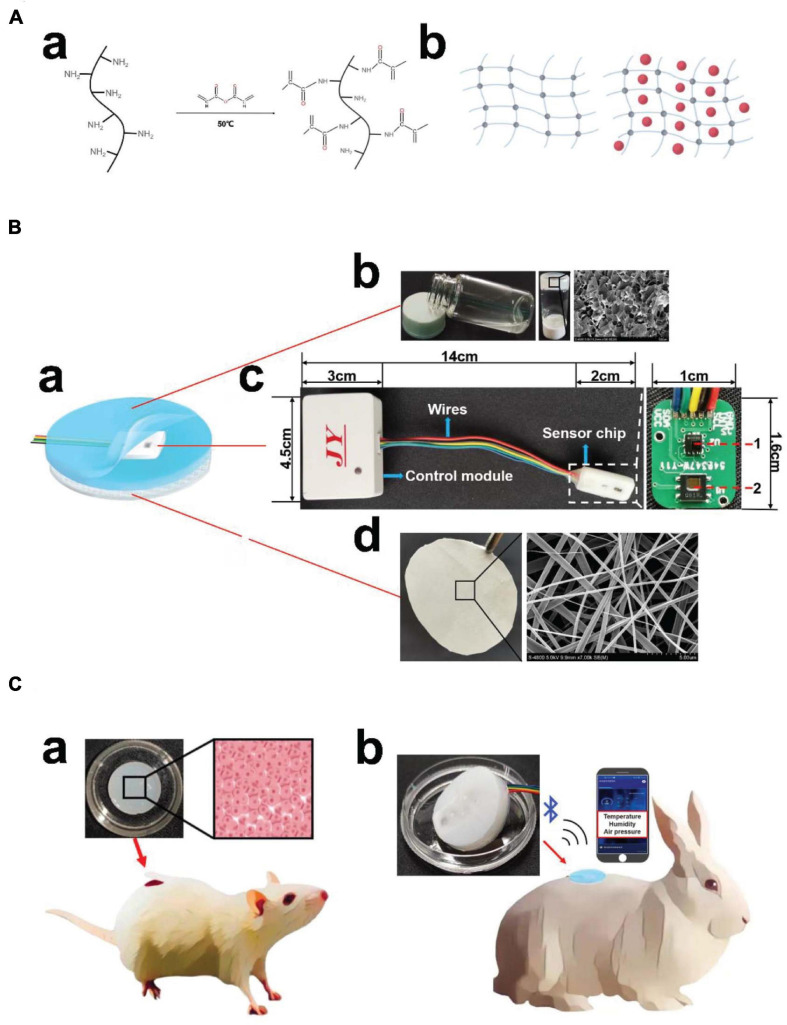
Structures and application scenario of the integrated smart dressing. **(A)** Schematic diagram of GelMA synthesis. **(a)** Gelatin undergoes a functionalization process using methacrylic anhydride with methacrylate and methacrylamide groups to form gelatine methacryloyl (GelMA). **(b)** Network structure of the GelMA hydrogel and β-cd in the GelMA hydrogel. **(B)** Schematic diagram of the integrated smart dressing structure. **(a)** The integrated smart dressing includes a microenvironment sensor, biomimetic nanofibre membrane and GelMA + β-cd UV crosslinked hydrogel. **(b)** Physical and detailed diagram of the prepolymer and crosslinked hydrogel. **(c)** Detailed diagram of the microenvironment sensor. (1, air-pressure sensor; 2, temperature and humidity sensor) **(d)** Photograph and detailed diagram of the biomimetic nanofiber membrane. **(C)** Physical diagram of the integrated smart dressing and schematic diagram of the animal experiment. **(a)** *In vivo* degradation and pro-angiogenic effect of the hydrogel. **(b)** *In vivo* monitoring and pro-healing effect of integrated smart dressings.

The smart dressing included three parts, namely, a biomimetic nanofibre membrane, sensor chip and hydrogel (Figure 1B). The temperature and humidity sensor and air-pressure sensor were welded on the sensor chip, which was connected to the control module outside the dressing through wires. The connecting part used a detachable port to facilitate replacement when there was no power. The control module included a BLE antenna, signal processing system, and battery. In addition, a mobile phone app was developed to receive, display, store and upload multiple groups of data after processing at the same time (Figure 1C). As smart dressings components, biomimetic nanofibre membranes and hydrogels could be replaced to meet different wound needs.

### Characterization of the GelMA-β-cd Hydrogels

**FIGURE 2:**
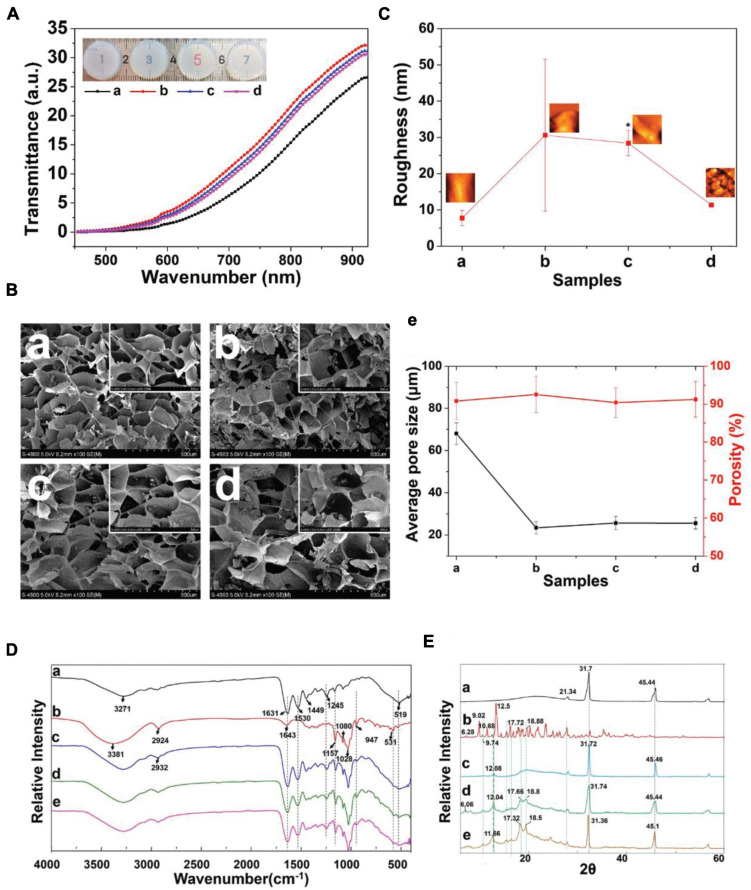
Characterization of the GelMA + β-cd UV crosslinked hydrogel. **(A)** Transmittance of different β-cd gradient hydrogels. (Label description: **(a)** pure GelMA hydrogel, **(b)** with 2% β-cd, **(c)** with 3% β-cd, **(d)** with 4% β-cd. The following are the same except for FTIR and XRD). **(Ba–d)** Surface morphological images of lyophilized crosslinked hydrogels examined by low- and high-magnification SEM. β-cd was evenly dispersed in the hydrogels and blended excellently with GelMA. **(e)** The porosity and pore size of the freeze-dried hydrogels obtained by mercury intrusion porosimetry. **(C)** Representative AFM images and the root mean square roughness (Rq) of different hydrogels observed in height mode. **(D)** FTIR spectra of panels **(a)** pure GelMA, **(b)** pure β-cd, **(c)** GelMA with 2% β-cd, **(d)** GelMA with 3% β-cd, and **(e)** GelMA with 4% β-cd. **(E)** XRD pattern of panels **(a)** pure GelMA, **(b)** pure β-cd, **(c)** GelMA with 2% β-cd, **(d)** GelMA with 3% β-cd, and **(e)** GelMA with 4% β-cd. (*P < 0.05 was considered statistically significant).

Figure 1A presents the GelMA hydrogel network and β-cd in the hydrogel. General images of the GelMA + β-cd gradient composite hydrogels are shown in Figure 2A. Overall, the light transmittance showed little difference among the groups of hydrogels. The transmittance of the GelMA + β-cd composite hydrogels was higher than that of the pure GelMA hydrogel and was the highest with the addition of 2% β-cd (w/v). Figure 2B shows FE-SEM images of both the surface and a cross-section of the freeze-dried GelMA + β-cd UV-crosslinked hydrogels at two different magnifications. Dense pores with a large size and uniform distribution were observed from the images of the pure GelMA hydrogel surface. After the introduction of β-cd, the interconnected 3D porous structure and uniformity of the freeze-dried gels were retained. Moreover, β-cd was evenly dispersed throughout the hydrogels and blended well with GelMA. No large difference between different β-cd amounts was observed. In addition, the porosity and pore size of the freeze-dried gels were determined by mercury intrusion porosimetry (Figure 2Be). A Bruker AFM instrument was utilized to determine the surface roughness of hydrogels, and the morphology of the GelMA + β-cd gradient composite hydrogels was examined. From the AFM analysis, the roughness of the pure GelMA hydrogel was 5-20 nm, while it increased to 15-45 nm after the addition of 2% or 3% β-cd (*p* < 0.001) (Figure 2C). When the content of β-cd was 2% or 3%, the surface roughness of the hydrogel was the highest, and it was similar to that of the pure GelMA hydrogel when the β-cd content was 4%.

The FT-IR spectra of GelMA, β-cd and GelMA + β-cd are shown in Figure 2D. The β-cd spectrum showed peaks at 3381, 1157 and 1028 cm^–1^, corresponding to O–H stretching vibrations, C–O asymmetric deformations and C–O–C symmetric stretching vibrations, respectively. All hydrogel spectra showed a broad peak, with a peak position at 3270 cm^–1^ associated with the stretching vibrations of the hydrogen-bonded hydroxyl groups. In the spectrum of the GelMA hydrogel derived from the modification of gelatine with MA, a strong peak appeared at approximately 1640 cm^–1^, primarily related to amide I C = O stretching groups. The band at 1500–1570 cm^–1^ corresponds to C–N–H bending vibrations, while the band at 3200–3400 cm^–1^ indicates the presence of peptide bonds (mainly N–H stretching vibrations). The peak at 1640 cm^–1^ corresponds to the carbon double bond in GelMA from the interaction between gelatine and MA. The spectra of hydrogels containing β-cd presented a similar increase in the same region. These results suggest an interaction between GelMA and β-cd. Diffractograms of the same compounds and inclusion complexes are illustrated in Figure 2E. GelMA showed obvious peaks at 21.34°, 31.7°, and 45.44°, while the β-cd crystalline profile was revealed by the presence of intense and narrow peaks. A comparison of the diffractogram of the GelMA + β-cd complex with that of β-cd indicated the disappearance of β-cd peaks at 9.02°, a shift of the peak at 11.66° to 12.08°, and a decrease in its intensity. The narrow and dense peaks of the original β-cd between 15° and 20° were replaced by two peaks at 17.7° and 18.8° and shifted to the left with increasing β-cd addition. The diffractogram of the hydrogel containing β-cd (GelMA + β-cd) presented similar peaks in the same region. These results show that a new crystal structure was formed between GelMA and β-cd. Interestingly, when the addition of β-cd was 2%, the shape of the peak was significantly different, which suggested that the addition of 2% β-cd may be a threshold for the formation of the crystal structure.

### Pro-angiogenic Effect of Hydrogels

**FIGURE 3:**
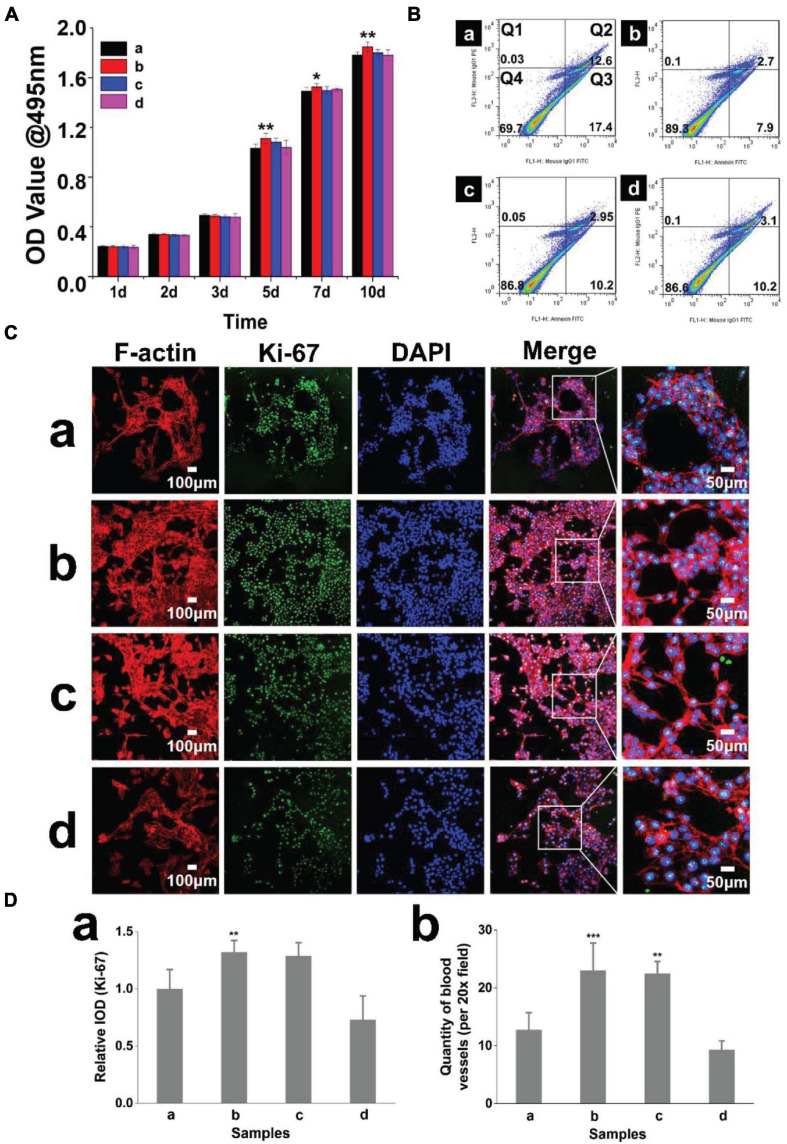
Hydrogel biocompatibility and promotion of lumen formation. **(A)** The CCK-8 method was used to quantify the cytotoxicity of the hydrogels, and the growth of cells cultured with extracts of the hydrogel with 2% β-cd was slightly better than that of the other three groups. (Label descriptions: **(a)** pure GelMA, **(b)** with 2% β-cd, **(c)** with 3% β-cd, and **(d)** with 4% β-cd.) **(Ba–d)** Flow cytometric quadrantal diagrams showing the effect of hydrogels on HDMEC apoptosis. The addition of β-cd could significantly reduce the apoptosis of HDMECs planted on hydrogels, but there was no significant difference between different amounts of β-cd. **(Ca–d)** Immunofluorescence images of HDMECs seeded on hydrogels (Ki-67, F-actin). HDMECs seeded on all samples showed obvious, well-ordered actin stress fibers. After the addition of β-cd, the lumen formation of HDMECs on hydrogels was significantly improved. **(Da,b)** Quantitative determination of the fluorescence intensity of Ki-67 and lumen number. On the hydrogels with 2 and 3% β-cd addition, the Ki-67 fluorescence intensity of HDMECs was the highest. The vascular tubule formation of HDMECs was significantly increased in the GelMA + β-cd group compared with the pure GelMA group. (*P < 0.05 was considered statistically significant, and **P < 0.01 and ***P < 0.001 were considered extremely significant).

To evaluate the biocompatibility of the hydrogels, we used a CCK-8 assay to analyze the cytotoxicity of the extracts. HDMECs cultured on all hydrogels exhibited a similar growth pattern of a time-dependent increase in cell number during the culture period. The activity of HDMECs on hydrogels (with 2% β-cd) was slightly higher than that of cells in the other groups (Figure 3A). We also analyzed the apoptosis of cells growing on the hydrogels and found that the apoptosis rate was the lowest when HDMECs were seeded on hydrogels with 2% β-cd (Figure 3B). Ki-67 immunofluorescence staining of the cells on hydrogels also verified the above results. Moreover, we labeled F-actin in primary HDMECs with a red fluorescent marker. As shown in Figure 3C, cells seeded on all samples showed obvious, well-ordered actin stress fibers. The number of tubules was analyzed by ImageJ software, and the results showed that the vascular tubule formation of HDMECs was significantly increased in the GelMA + β-cd group compared with the pure GelMA group (Figure 3D). Additionally, the cells converged more tightly, and the tubules became thicker in the 2% and 3% β-cd groups than in the other groups, while the tubules formed in the pure GelMA group were crumblier and broke more easily. In summary, we decided to use a GelMA hydrogel with a 2% β-cd content for the follow-up experiments.

**FIGURE 4:**
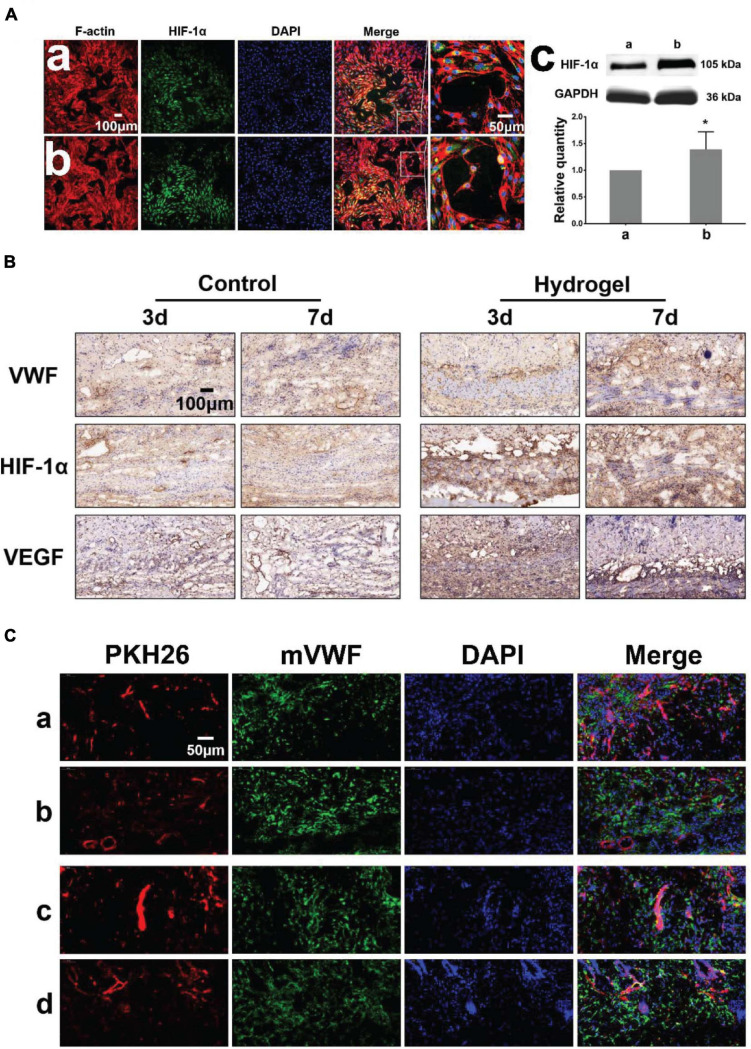
Pro-angiogenic effect of hydrogels *in vitro and vivo*. **(Aa,b)** Immunofluorescence images of HDMECs implanted on blank confocal dishes and 2% β-cd hydrogel (HIF-1α, F-actin). **(c)** Western blot analysis and quantification of HIF-1α expression of HDMECs on the hydrogel. The expression of HIF-1α in HDMECs on the 2% β-cd hydrogel was significantly higher than that in HDMECs on blank confocal dishes. **(B)** Immunohistochemical images of subcutaneously implanted hydrogels (VWF, HIF-1α, VEGFA). The hydrogel group formed a neovascularization network at the initial stage of implantation and gradually matured, while the control group lagged behind. The hydrogel group showed high HIF-1α and VEGFA expression in the new granulation area compared with the control group. **(C)** Immunofluorescence images of subcutaneously implanted hydrogels (PKH26, mVWF, DAPI). The HDMECs loaded in the hydrogel survived normally at the initial stage of implantation in mice and gradually formed new vessels with mouse tissue cells. [Label descriptions: **(a)** longitudinal section of 3 days, **(b)** cross section of 3 days, **(c)** longitudinal section of 7 days, **(d)** cross section of 7 days]. (*P < 0.05 was considered statistically significant).

We detected the expression of hypoxia-inducible factor-1α (HIF-1α) when HDMECs on the hydrogel (with 2% β-cd) formed a large number of lumens (Figures 4Aa,b). Western blot analyses showed greater HIF-1α protein expression in the hydrogel group than in the control group (Figure 4Ac), consistent with the cellular immunofluorescence results. To study the degradation and pro-angiogenic effect of hydrogels *in vivo*, hydrogels loaded with PKH26-labeled HDMECs were implanted subcutaneously in BALB/c mice and the tissue was stained by immunofluorescence and immunohistochemistry on the 3rd and 7th days. The results showed that a vessel network began to form on the 3rd day and improved gradually to the 7th day in the hydrogel group. However, in the control group, vessel network formation was not observed on the 3rd day (Figure 4B). There was more neovascularization around the hydrogels than in the control group, and the expression of HIF-1α and VEGF was upregulated in the tissues, which indicated that the hydrogel could promote tissue angiogenesis to a certain extent. The results of immunofluorescence staining for mouse wounds showed that at the early stage of implantation, the cells loaded on the hydrogel could survive in mice, and over time, these cells and mouse cells formed new blood vessels together (Figure 4C).

### Characteristics and *in vivo* Application of Smart Dressings

**FIGURE 5:**
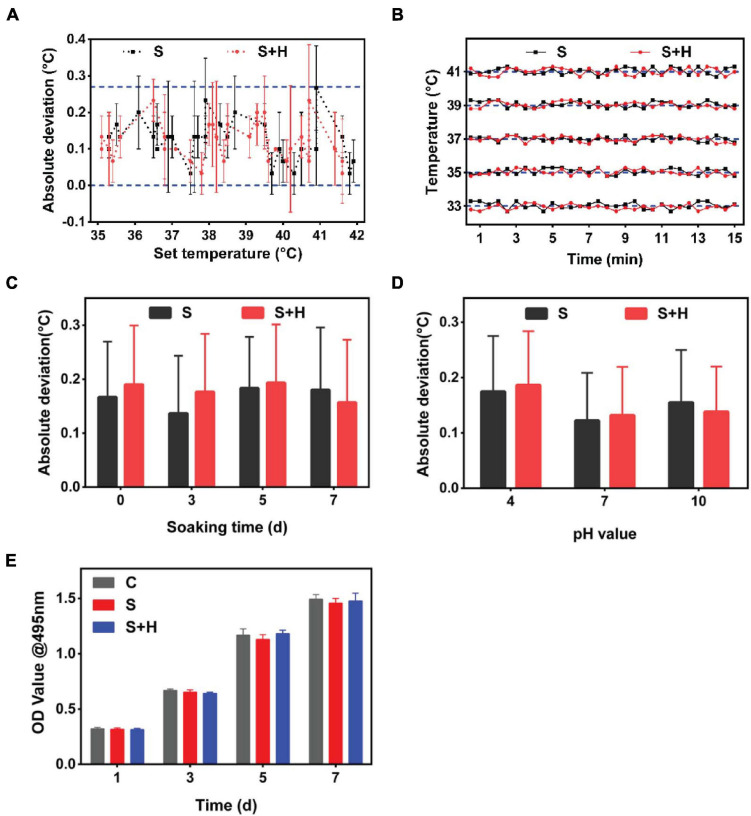
*In vitro* operation of the integrated smart dressing and its accuracy, stability, biological durability and biocompatibility. **(A)** Monitoring accuracy of the temperature sensor in the integrated smart dressing, which was defined as the deviation between the recorded average temperatures and the corresponding setting temperatures in the range of 33 ∼ 41°C. (Label descriptions [here and below]: C, Control; S, Sensor chip; S + H, Sensor chip + Hydrogel.) **(B)** Monitoring stability of the temperature sensor in the integrated smart dressing. This information was profiled by the time-temperature curves recorded continuously for 15 min at different setting temperatures. **(C)** Durability of the temperature sensor in the integrated smart dressing, which is presented as the absolute deviation compared with the setting temperature after being soaked in DMEM for different times. **(D)** Measurement accuracy of the integrated smart dressing after soaking in different pH buffers for 3 days. In the buffer solution of pH 4, the measurement deviation is slightly higher, but the overall deviation is within 0.2°C. **(E)** Biocompatibility of the integrated smart dressing, which was evaluated by the viability of L-929 cells cultured in the extract solution. Cells cultured in DMEM served as controls (*n* = 8).

According to the mean absolute deviation curve, the smart dressing exhibited satisfactory accuracy, with a deviation of mostly < 0.3°C (Figure 5Aa, blue dotted line). As shown by the temperature–time curve (Figure 5B), the smart dressing displayed a short response time of < 30 s and good long-term stability. Considering that the working environment of the system was complicated, the durability of the smart dressing was evaluated by immersing it in buffer with different pH values and endothelial cell medium for different time intervals at 37°C. There was no significant change in the measurement deviation within 5 days (Figures 5C,D). We evaluated the cytotoxicity of the smart dressing using HDMECs as a model. HDMECs were cocultured with the extract solution of the smart dressing for 1, 3, 5, and 7 days. Cells cultured with endothelial cell medium were used as a control. Cell viability was assessed at each time interval using the CCK-8 assay. There was no significant difference in the absorbance of the culture medium among the samples (Figure 5E).

**FIGURE 6:**
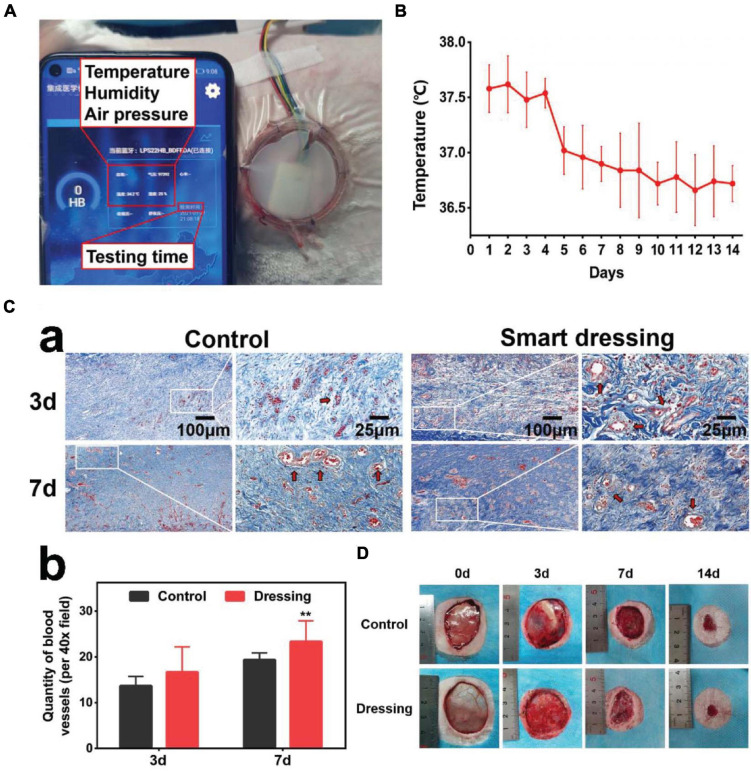
*In vivo* application of the integrated smart dressing. **(A)** The integrated smart dressing enables the real-time monitoring of the microenvironment of wounds. **(B)** Characteristics of local temperature changes during wound healing recorded by the smart dressing. **(C)** Masson staining results of two groups of wound samples. **(a)** The wounds bandaged with smart dressings showed faster and higher quality neovascularization and a reduced inflammatory reaction than those in the control group (red arrow: neovascularization). **(b)** Quantitative analysis of neovascularization. **(D)** Images of the wound healing process. The wound healing of the integrated smart dressing was faster than that of the control. (**P < 0.05 was considered extremely significant).

Smart dressings enabled real-time monitoring of the wound temperature (Figure 6A). The trend of change in the wound temperature is shown in Figure 6B. By observing the temperature change curve of the wound, we found that the local temperature was slightly high on the first 3-4 days, which may represent the stage of hyperemia and inflammation of wound healing. As the wounds entered the repair stage, the local temperature decreased and reached a stable plateau phase. The Masson staining results showed that the wounds bandaged with smart dressings showed more obvious neovascularization and less inflammation than those in the control group on the 3rd and 7th days (Figure 6C). The photographs showed that the wound healing rate in the smart dressing group was faster than that in the control group (Figure 6D).

## Discussion

When and whether a dressing should be changed are currently not standardized. The wound state should be used to guide dressing changes, however, this information can currently only be obtained by opening the dressing to observe the wound. This traditional wound evaluation method lacks an objective basis and relies heavily on the level and clinical experience of the attending physician. However, repeated dressing changes, not only interfere with the normal wound healing process but also increase the risk of wound infection (Collier and Hollinworth, 2000; Powers et al., 2016). Moreover, most of the active ingredients used in debridement, such as hydrogen peroxide and ionic silver preparations, are locally toxic and demonstrate limited or no proven efficacy in enhancing wound healing (Robert et al., 2013). In sudden situations, such as wars and natural disasters, when a serious shortage of medical resources emerges, it is difficult for medical staff to change dressings in a timely manner, which may delay treatment and lead to wound deterioration. More importantly, wound healing should be evaluated throughout the treatment process, but traditional treatment methods based on clinical experience cannot meet this requirement (Marieke, 2018). The clinical values of various cells and cytokines near the wound bed were found to much lower than previously expected (Sabin and Richard, 2003; Patel et al., 2016). In addition, the disadvantages of traditional clinical experience-based therapy, including infection and medical resource abuse, have been revealed in clinical practice. In the event of large-scale public health events, such as the coronavirus disease 2019 (COVID-19) pandemic, failure to change a dressing in isolated patients a timely manner could contribute to wound deterioration. The evaluation of clinical outcomes, which guides real-time monitoring and highly effective medical interventions, plays an essential role throughout wound treatment. It has been established that four phases comprise the healing process: hemostasis, inflammation, proliferation and tissue remodeling or resolution (Morton and Phillips, 2016). Recent studies have demonstrated that the microenvironment of wounds differs in each stage of healing and under conditions of infection by different pathogens (Kruse et al., 2015). Therefore, we designed an integrated smart dressing with a three-layer structure that could monitor the wound microenvironment in real time while maintaining the biological function of dressings without disturbing the healing process, providing a promising scheme for intelligent wound care.

The ECM-like structure of hydrogels plays an important role in physiological processes such as cell growth, differentiation, apoptosis and gene expression. Moreover, certain aspects of 3D GelMA hydrogels closely resemble some essential properties of the native ECM due to the presence of cell attachment and matrix metalloproteinase-responsive peptide motifs, which allow cells to proliferate and spread in GelMA-based scaffolds (Shin et al., 2012; Zhao et al., 2016). Such materials undergo crosslinking when exposed to light irradiation to form hydrogels with tuneable mechanical properties that mimic the native ECM (Salber et al., 2007; Trappmann et al., 2012). β-cd, a common additive, is often used to improve the physical structure, mechanical properties and biocompatibility of biomaterials. However, some studies have shown that an excessively high concentration of β-cd can inhibit the growth of all cell lines in a dose-dependent manner, so it is important to determine the appropriate concentration of β-cd in hydrogels (Niu et al., 2018; Bezerra et al., 2020). It is known that the microstructure, including the pore size and its distribution, has a prominent influence on cell intrusion, proliferation and function in tissue-engineered hydrogels. The nearly transparent appearance and porous microstructure ensure that the hydrogel has good visibility and water absorption ability. The FT-IR results confirm that with the occurrence of complexation, β-cd is successfully intercalated and deposited. The change in the diffraction peak in the XRD results confirms the formation of a new crystal structure (Rahali et al., 2017). Interestingly, 2% β-cd appears to be a threshold for forming this crystal structure, which is also reflected in the AFM results. Porous microstructures and relatively rough surfaces are also more convenient for cell adhesion and crawling. The swelling ratio (SW) of hydrogels determines their exudate-absorbing capacity. The SW of hydrogels is significantly dependent on the amount of β-cd. In addition to the FE-SEM and mercury porosimetry results, the pore size of the hydrogels decreased with the addition of β-cd, but the change in porosity was not obvious. This phenomenon could be explained in terms of the internal structure and crosslinking degree of the hydrogels. Moreover, the SW of the prepared hydrogels was associated with the crosslinking degree, which affected the water absorbency of the hydrogels, except for UV crosslinks. Physical crosslinking of the polymer chains plays an important role in the SW of the hydrogels (Raghuwanshi and Garnier, 2019). With increasing β-cd concentration, physical crosslinking of the hydrogels was strengthened by more intermolecular hydrogen bonds and chain entanglements between β-cd and GelMA, leading to a lower swelling degree with increasing β-cd content. The alternative view is that in this case, β-cd played a physical crosslinker role, and its addition enhanced the crosslinking density by increasing the number of crosslinking sites, inducing a decrease in the SW (Sherje et al., 2017). At the same time, the change in structure caused by β-cd is also responsible for the change in hydrogel water uptake. With the increase of external pH value, the degree of protonation of hydrogels increases, which enhances their hydrophilicity and swelling capacity. In a neutral environment, protonation and deprotonation reach a balance, so the hydrogel can remain stable in a simulated human environment (Legros et al., 2017; Ehtesabi et al., 2020). In addition, an acidic environment may be more conducive to hydrogels degradation. The results of the SWE and WCA tests showed the good softness and hydrophilicity of the hydrogels, demonstrating that the hydrogels could protect and moisten the wound surface and supporting their application *in vivo*.

Good biocompatibility is a prerequisite for hydrogels to play various biological roles (Theoret, 2005). The results of our research demonstrate the suitability of GelMA hydrogels for cell proliferation due to their innate biocompatibility. The appropriate addition of β-cd significantly improved the biocompatibility of the GelMA hydrogel and reduced the apoptosis of cells growing on the hydrogel. New vessel formation in the wound area is essential for the wound healing process. This process removes necrotic tissues and supports nutrition in the wound bed. VECs are subjected to shear stress caused by blood circulation for a long time; thus, external mechanical signals may affect the morphology and function of blood vessels by regulating the biological behavior of VECs. In this study, a tubule formation assay was used to investigate the effect of hydrogels on the tubule formation of HDMECs. As a result, the HDMECs showed obvious, well-ordered actin stress fibers and good lumen-forming behavior on all kinds of hydrogels. The greatest number of lumens formed on the GelMA hydrogel with 2% β-cd, which is in accordance with the AFM and XRD results. Therefore, we chose this hydrogel concentration for follow-up experiments and performed a preliminary study on the related mechanism. HIF-1α, part of a well-studied angiogenesis pathway, induces the expression of some cytokines associated with vascular development, such as VEGF, which has been confirmed to increase endothelial cell proliferation, survival, and migration and promote angiogenesis (Dorit et al., 1992; Hong et al., 2014). Previous studies showed that HIF-1α was highly expressed in the wound beds during the early stage of the normal wound healing process (Xing et al., 2011). However, in chronic wounds, impairment of HIF-1α transactivation was due to coactivator p300, induced by reactive oxygen species (ROS), resulting in less vessel network formation (Jaakkola et al., 2001). The high expression of HIF-1α in the tissue near implanted hydrogels also indicates its potential application in chronic wound repair. Regarding tissue engineering hydrogels, the degradation rate is also an important factor affecting the rate of new tissue formation. From this point of view, once new tissue forms, the hydrogels should be completely degraded and absorbed by the body. Based on the experimental data from the degradation behavior of various hydrogels *in vitro* and *in vivo*, the hydrogels were almost completely degraded, and only a few implanted hydrogel fragments were distributed throughout the loose connective tissue at the implantation site after 3 weeks. As the wound would reach the remodeling period during the second week after transplantation, we decide to collect tissue samples on the 3rd and 7th days. Researches have shown that the implanted vascular networks anastomose with host vessels through a process of “wrapping and tapping” between the engrafted endothelial cells and the host vasculature (Cheng et al., 2011). The research showed that the HDMECs loaded on the hydrogel survived normally during the initial stage after implantation in mice and gradually formed new vessels with mouse cells, and the growth of mouse vessels near the wound area supported further integration with the implanted human vasculatures, which played a certain role in promoting angiogenesis (Hanjaya-Putra et al., 2013; Morini et al., 2018; Delgado-Bellido et al., 2019). Early vessel formation plays an important role in wound healing. In this study, an immunohistochemical staining method for endothelial cells was used to investigate the effect of GelMA + β-cd hydrogels on early vessel formation at the implant site. We found that HIF-1α expression was upregulated in endothelial cells and dermal fibroblasts near the implanted hydrogels. Fibroblasts play a core role in the process of wound reconstruction, and the upregulation of HIF-1α expression in fibroblasts could significantly increase the expression of angiogenesis-related cytokines, such as VEGF, promoting new vessel formation in the wound area (Chen et al., 2016). With vessel ingrowth, more nutrients and other growth factors were transferred to the wound area and enhanced the recovery function of fibroblasts and other cells. The application of hydrogels on the wounds of rats also proved that the hydrogel could promote the healing of chronic wound of diabetes.

When designing integrated smart dressings, complex conditions, such as the wound temperature range and liquid environment, should be taken into account. The accurate perception of the wound by microenvironment sensors is based on good measurement accuracy and stability, which determines whether the measurements can truly reflect the changes in the measured parameters to infer the real-time state of the wound. In our study, microenvironment sensors combined with biological dressings showed good measurement accuracy, stability and durability. The accuracy of the temperature measurement was no less than that in previous studies (>0.3°C) (Potyrailo et al., 2011). Smart dressings are in direct contact with wound tissue and cells, so they should neither be cytotoxic nor negatively affect cell growth. Cytotoxicity tests also confirmed the good biocompatibility of the integrated smart dressings. Through the use of an integrated smart dressing to visualize the wound microenvironment, it is more convenient for clinicians to evaluate the state of wound healing to make correct treatment decisions. The local wound temperature is often considered a predictor of infection (Tegl et al., 2015; Power et al., 2017). When the temperature of a wound is less than 33°C, wound repair is hindered (Kurz et al., 1996; McGuiness et al., 2004). The local temperature of acute wounds may be greater than 37°C due to local congestion and inflammation; however, a sudden increase in wound temperature is regarded as a sign of infection (Fierheller and Sibbald, 2010). Abundant microenvironmental parameters can indicate the state of the wound and, thus, are helpful tools for the non-invasive monitoring and early detection of wound non-healing or infections (Kruse et al., 2015). In addition to temperature, several parameters, including the local pH, glucose, lactic acid, and uric acid levels, may represent available candidates for subsequent research. We believe that the richer the microenvironment index is, the more accurate the estimate of the wound state. Furthermore, we can explore a more in-depth combination of a microenvironment monitoring system and functional biological dressings, such as the establishment of a remote wound intervention system controlled by mobile terminals based on drug-loaded hydrogels. While judging the wound state by the wound microenvironment, clinicians can remotely control the dressing to manipulate the wound-healing process. The ideal smart dressing should include smart monitoring, smart and smart control functions (Farahani and Shafiee, 2021). At present, our research only realizes smart monitoring function. Future work could improve smart dressings through increasing the richness of monitoring indicators, improving wound clinical outcome algorithms, developing more intelligent medical treatment programs for casualties and performing data analysis regarding the distribution and severity of acute or chronic wounds. We believe that intelligent and individualized wound care and diagnosis based on the wide application of “big data” and the Internet of Things represents the future of clinical wound management.

## Conclusion

In this study, we demonstrate the capability of an integrated smart dressing composed of a biomimetic nanofibre membrane, microenvironment sensor and GelMA + β-cd UV crosslinked hydrogel for wound microenvironment monitoring and promoting angiogenesis and wound healing. The hydrogel could significantly accelerate neovascularization, granulation tissue remodeling and epithelial crawling *in vivo* by increasing the expression of HIF-1α, and the sensor exhibited good measurement accuracy, stability, biological durability and biocompatibility. The integrated smart dressing not only enables the non-invasive real-time monitoring of the wound microenvironment but also retains the biological function of tissue engineering dressings. It has potential for clinical application and may play a significant role in telemedicine, AI-based diagnosis and precision medicine.

## Data Availability Statement

The raw data supporting the conclusions of this article will be made available by the authors, without undue reservation.

## Ethics Statement

The animal study was reviewed and approved by the Fourth Military Medical University Tangdu Hospital Ethics Committee.

## Author Contributions

XL, TL, and JL did the conception of the study. YZ and CZ wrote the original draft of the manuscript and carried out the experimental performance. RH, TL, and XL wrote and revised the manuscript. QZ, YL, and JL performed the analysis. All authors contributed to the article and approved the submitted version.

## Conflict of Interest

The authors declare that the research was conducted in the absence of any commercial or financial relationships that could be construed as a potential conflict of interest.

## Publisher’s Note

All claims expressed in this article are solely those of the authors and do not necessarily represent those of their affiliated organizations, or those of the publisher, the editors and the reviewers. Any product that may be evaluated in this article, or claim that may be made by its manufacturer, is not guaranteed or endorsed by the publisher.

## Supplementary Material

Supplementary Figure 1 General photographs and ultrastructure of the GelMA prepolymer, swelling and water uptake of the GelMA + β-cd hydrogel. **(A)** The GelMA prepolymer was a white spongy object with a closed-pore lamellar structure on the surface **(a)**. The cross-section showed a loose porous structure alternating vertically and horizontally **(b)**. **(Ba)** Physical diagram of hydrogels in different states (State 1. Initial; State 2. Swelling; State 3. Dehydration; State 4. Reswelling). **(b)** Swelling ratio and water intake capacity of each hydrogel. The swelling ratio of the prepared composite hydrogels decreased with increasing β-cd content, and the water uptake showed the opposite trend. [Label descriptions: **(a)** pure GelMA, **(b)** with 2% β-cd, **(c)** with 3% β-cd, and **(d)** with 4% β-cd].

Supplementary Figure 2 Swelling of hydrogels in solutions with different pH values or NaCl concentrations and *in vitro* degradation. **(A)** Swelling of hydrogels in different pH solutions. When the hydrogels were immersed in different pH buffers at 37°C for 4 weeks, the weight of the hydrogels did not change significantly when the pH value was 7, but decreased when the pH value was 4, and increased significantly when the pH value was 10. **(B)** Swelling of hydrogels in solutions with different NaCl concentrations. At different concentrations of NaCl solution at 37°C for 4 weeks, the swelling ratio of the hydrogels increased with increasing β-cd content. [Label descriptions: **(a)** pure GelMA, **(b)** with 2% β-cd, **(c)** with 3% β-cd, and **(d)** with 4% β-cd].

Supplementary Figure 3 Degradation of hydrogels *in vitro* and *in vivo*. **(A)** Degradation of hydrogels in 2 mg/mL type I collagenase solution. The mass of all hydrogels first increased and then decreased and basically degraded within 5 days. With the increase in the amount of β-cd, the degradation rate of the hydrogel increased *in vitro*. On the 5th day, a small amount of pure GelMA hydrogel remained, and that in the other groups had been completely degraded. [Label descriptions: **(a)** pure GelMA, **(b)** with 2% β-cd, **(c)** with 3% β-cd, and **(d)** with 4% β-cd.] **(B)** Degradation of the hydrogel *in vivo*. With the increase in implantation days, the volume of subcutaneous residual hydrogels decreased, and they are almost completely degraded on the 21st day. **(a–e)** represent days 0, 3, 7, 14, and 21, respectively.

Supplementary Figure 4 Shear wave elastography (SWE) and water contact angle (WCA) results of hydrogels. **(A)** Young’s modulus of hydrogels. The hydrogels in all groups were soft, and the Young’s modulus of hydrogels increased with increasing β-cd content. **(B)** Hydrophilicity of hydrogels. All groups of hydrogels were hydrophilic, and the hydrophilicity decreased with increasing β-cd content. [Label descriptions: **(a)** pure GelMA, **(b)** with 2% β-cd, **(c)** with 3% β-cd, and **(d)** with 4% β-cd].

Supplementary Figure 5 The key port of the control module, the different working states of the sensor and the construction process of the integrated smart dressing. **(A)**. The key interface of the control module of the integrated smart dressing. **(B)** Different working states of the microenvironment sensor *in vitro*. **(a)** Signal search. **(b)** Upon connection. **(c)** Display interface. **(d)** Turn off the switch. **(C)** The manufacturing process of the integrated smart dressing. **(D)** Integrated smart dressings composed of hydrogel and sensor chips could still enable real-time monitoring of wound microenvironment parameters. **(E)** The process of the application of integrated smart dressing on wounds of full-thickness skin defects on the back of rabbits. The wound was established and fixed with a plastic ring. A nanofibre membrane was applied to the wound, and the hydrogel combined with the sensor was then placed on the wound.

Supplementary Figure 6 Three-dimensional image of lumen formed by HDMECs seeded on hydrogels. The three-dimensional lumen-like structures were formed in all groups, and the best morphology was in b and c group, which formed a vascular-like network. [Label descriptions: **(a)** pure GelMA, **(b)** with 2% β-cd, **(c)** with 3% β-cd, and **(d)** with 4% β-cd].

Supplementary Figure 7 Application of hydrogels in chronic wounds. **(A)** Images of the wound healing process of diabetic rats. The healing speed of normal or hydrogel-treated wounds was significantly faster than that of diabetic rat wounds, and the hydrogel-treated group was even the fastest. **(B)** Immunohistochemical images of the wounds of diabetic rats (VWF). Only a small amount of abnormal angiogenesis could be seen in diabetic wounds. On the 7th day, a network of new small blood vessels was formed on normal wounds. The rate of new blood vessels forming was faster and the morphology was better after the application of hydrogel. (Label descriptions: **(a)** diabetic wound, **(b)** normal wound, and **(c)** diabetic wound treated with the hydrogel).

Supplementary Table 1 pH value of hydrogels before and after gelation with different β-cd amount (%).
