# Supplementary material for: An Integrated Smart Sensor Dressing for Real-Time Wound Microenvironment Monitoring and Promoting Angiogenesis and Wound Healing
Source: Front Cell Dev Biol. 2021 Aug 6;9:701525. doi: 10.3389/fcell.2021.701525 (PMC8378138; doi:10.3389/fcell.2021.701525)
Supplement: Supplementary Figure 1 — General photographs and ultrastructure of the GelMA prepolymer, swelling and water uptake of the GelMA + β-cd hydrogel. (A) The GelMA prepolymer was a white spongy object with a closed-pore lamellar structure on the surface (a). The cross-section showed a loose porous structure alternating vertically and horizontally (b). (Ba) Physical diagram of hydrogels in different states (State 1. Initial; State 2. Swelling; State 3. Dehydration; State 4. Reswelling). (b) Swelling ratio and water intake capacity of each hydrogel. The swelling ratio of the prepared composite hydrogels decreased with increasing β-cd content, and the water uptake showed the opposite trend. [Label descriptions: (a) pure GelMA, (b) with 2% β-cd, (c) with 3% β-cd, and (d) with 4% β-cd]. [file Data_Sheet_1.pdf]

Supplementary materials

1. Materials and methods

1.1 Materials

Gelatin (Type A from porcine skin), β-cd (97%), methacrylic anhydride (MA), 2-hydroxy-4' (2-hydroxyethoxy) v-2-methylpropiophenone were purchased from Sigma Aldrich (St. Louis, MO, USA). Phosphate buffered saline (PBS) and Dulbecco's phosphate buffered saline (DPBS) were obtained from HyClone (Logan, Utah, USA). All other materials and reagents were of analytical grade. Deionized (DI) water (≥18.2 MΩ) from a Milli-Q system (Millipore, USA) was used in all aqueous solutions.

1.2 Synthesis of GelMA prepolymer

The synthetic method of GelMA is improved from the previous study(Bulcke et al., 2000). In brief, 10 g of type A porcine skin gelatine was dissolved in 100 mL of DPBS at 50°C under constant stirring. Under heating and stirring, 8 mL of MA was added at a rate of 0.5 mL/min. The mixture was placed in a 50°C shaker, and after 3 h, 400 mL of DPBS was added to stop the reaction. Maintaining a higher pH during the reaction enhances the reactivity of amine and hydroxyl groups, thereby leading to a higher degree of substitution(Hoch et al., 2012). The mixture was packed in dialysis bags (MWCO 12-14,000, Fisher Scientific) and immersed in plenty of 37°C DI water for 1 week to remove salts and other small molecule impurities. The DI water was changed every 12 h. The solution was then frozen overnight at -80°C and freeze-dried for 1 week. The resulting white sponge was stored at -80°C until later use.

1.3 Swelling behavior and water uptake study

First, cylindrical disc specimens (approximately 15 mm in diameter; 7 mm thick) were cut

from purified UV-crosslinked GelMA+β-cd gradient composite hydrogels, photographed. Second, the hydrogel discs were immersed in PBS at 37°C with reciprocal shaking (80 rpm) in a thermostatically controlled water bath to reach equilibrium swelling. Third, the swollen hydrogel discs were removed from the swelling medium and dehydrated with absolute ethanol. Finally, the dehydrated samples were immersed in PBS solution for rehydration. The weights of the prepared hydrogel discs in the initial, swollen, dehydrated and reswollen states were recorded as Wi, Ws, Wd and Wrs, respectively. In addition, the gross appearances of the hydrogel discs were photographed by a digital camera (Nikon, Melville, NY, USA), with the diameter and thickness recorded. The equilibrium swelling rate (ESR) and water uptake (WU) of the test hydrogels were obtained from equations (1) and (2), respectively:

$$ESR\ (\%) = Ws/Wd \times 100\% \quad (1)$$

$$WU\ (\%) = (Wrs-Wd)/Ws \times 100\% \quad (2)$$

1.4 The stability test of hydrogels under physiological conditions

To further evaluate the swelling kinetic responsiveness of the prepared GelMA+β-cd gradient composite hydrogels to various pH values and ionic strengths, a certain amount of lyophilized hydrogel discs (Wd) was placed into a 50 mL centrifuge tube containing 10 mL of PBS at various pH values (4.0, 7.0 and 10.0) and sodium chloride solution at different concentrations (0.01, 0.02, 0.03, 0.04, 0.05, 0.06, 0.07 and 0.08 mg/mL) and incubated at 37°C on a rotary shaker (150 rpm). At certain time periods (10 min, 20 min, 30 min, 1 h, 6 h, 12 h, 24 h, 2 d, 3 d, 5 d, 6 d, 1 week, 2 weeks, 3 weeks, and 4 weeks), the wet weight of each time point (Wt) of each sample was determined after wiping off excess solution on the surfaces using Kimwipes. The buffer was refreshed after each weighing, and the measurements were performed in triplicate. Then, the

swelling ratio (SW) was calculated according to the following equation:

Swelling ratio = Wt/Wd×100% (3)

1.5 *In vitro* degradation study

The degradability of the UV-crosslinked GelMA+β-cd gradient composite hydrogel discs was evaluated by measuring the change in weight of the gels at regular time intervals after incubation with type I collagenase at 37°C. In brief, a certain amount of each test hydrogel sample was first weighed (Wi) and immersed in 5 mL of PBS containing 2 mg/mL type I collagenase (9001-12-1, Gibco) in PBS at 37°C with reciprocal shaking (100 rpm) in a thermostatically controlled water bath for up to 3 weeks. The samples were subsequently removed and washed with double distilled water (DDW) at predetermined time intervals. The solution was replenished after each weighing, and the degraded samples were collected by filtration and further dried in vacuo. The weights of the samples at predetermined time points (0, 5 min, 10 min, 20 min, 30 min, 40 min, 50 min, 60 min, 2 h, 4 h, 6 h, 12 h, 1 d, 2 d, 3 d, 4 d, and 5 d) were determined. All the experiments were performed in triplicate.

1.6 Measurement of Young's modulus

In this paper, shear wave elastography (SWE) was applied to determine the hyperelastic parameters of hydrogels that exhibited stiffness similar to that of soft skin. SWE measurements were performed by using a Mindray Resona 7 diagnostic ultrasound system (Mindray, Shenzhen, China) with an L11-3U linear array transducer (5.6-10.0 Hz). The prepared samples were placed between palmar skin and isolated pig skin and detected with a transducer. The Young's modulus of the prepared hydrogel was initially measured in SWE mode in a uniform state.

1.7 Measurement of water contact angle

The contact angle (CA) is defined as the angle between the baseline of a liquid droplet and the

tangent line at the point of contact of this water droplet with the surface. The CA of water in air on the hydrogel surface was measured using a Dataphysics DCAT 11 (Dataphysics instruments, Germany) after a water droplet of approximately 10 μL was placed on the surface of the hydrogel using a micro-syringe. All hydrogel samples (3×10 cm) were glued on a movable sample stage (black Teflon-coated steel, 7×11 cm) that was levelled horizontally before measurement.

1.8 Pro-healing and pro-angiogenesis effects of the hydrogel in chronic wounds

A total of 9 male SD rat (300±50 g) were used in this study. 3 of them were normal and 6 were diabetic induced by STZ(Reed et al., 2000). All surgery was performed under anaesthesia. After anaesthetization with 1% pentobarbital sodium for 5 min, the hairs on the backs of the rats were shaved. A standardized 1 cm diameter full-thickness wounds were created on the middle of the rat' s back and a metal ring was fixed around the wound to prevent contraction. The wounds of 3 normal rats and 3 diabetic rats were bandaged routinely with gauze. The wounds of the other 3 diabetic rats were covered with hydrogels (with 2% β-cd) and fixed with gauze. After surgery, the animals were given antibiotics twice daily for 48 h. The wound area was recorded by digital camera on the 0th, 3rd, 7th and 14th days after modelling, and the samples were collected on the 3rd and 7th days. Three rats were used for each data point. The obtained wound specimens in each group were fixed in 4% paraformaldehyde at 4°C overnight, dehydrated with a graded series of ethanol, embedded in paraffin and sliced.

1.9 Immunofluorescence and immunohistochemical staining

After indicating a lumen-like morphology of the HDMECs at day 5, the cell-laden hydrogel structures were fixed with PBS containing 4% (v/v) formaldehyde for 24 h at 4°C followed by three washes in PBS for 5 min each. Subsequently, the samples were permeabilized with 1% (w/v) bovine

serum albumin (BSA) in PBS containing 0.1% (w/v) Triton X-100 for 45 min at RT. After blocking, the samples were incubated with anti-Ki-67 (Affinity, AF-0198, 1:100) and anti-HIF-1α (Affinity, AF-1009, 1:100) overnight at 4°C and counterstained with goat anti-rabbit IgG (H+L) Fluor488-conjugated secondary antibodies (Affinity, S0018, 1:100), phalloidin-iFluor 594 (1:1000, ab176757, Abcam) and 4',6-diamidino-2-phenylindole (DAPI, 1:500, 28718-90-3, Sigma Aldrich) at RT. Then, the optimal preparation conditions of the hydrogels were determined based on the lumen number and proliferation data and further applied for the subsequent experiments.

The endogenous peroxidase of randomly selected mouse sections was inactivated by incubation with 3% H_2O_2 in PBS for 20 min. To recover antigen, these sections were placed into 10 mM sodium citrate buffer solution (pH 6.0) and heated in a microwave oven for 15 min, and the slides were allowed to cool at RT and then were washed three times with PBS for 3 min each. The non-specific binding sites were blocked with 5% goat serum (Gibco) in a humid chamber for 30 min at RT. After the excess liquid was discarded, the sections were incubated with the primary antibodies anti-VWF (Affinity, AF-3000, 1:100), anti-VEGFA (Proteintech, 19003-1-AP, USA) and anti-HIF-1α (Affinity, AF-1009, 1:100) at 4°C overnight; next, they were washed with PBS, followed by incubation with a biotinylated goat anti-rabbit secondary antibody kit (Beyotime Biotechnology, China) at 37°C for 30 min and then incubation with streptavidin-HRP for 30 min. The antibody binding sites were visualized by incubation with a 3,3'-diaminobenzidine (DAB) solution. The slides were counterstained with haematoxylin and then dehydrated with sequential ethanol washes for sealing and microscope observation. The same method was used for immunohistochemical staining of rat sections. For immunofluorescence staining, after incubation with anti-VWF (Affinity, AF-3000, 1:100) using the same method, the samples were incubated with

goat anti-rabbit IgG-488 conjugate secondary antibody for 30 min at RT in the dark, and cell nuclei were stained with DAPI. Subsequently, the samples were washed with PBS, mounted with anti-fade mounting medium (Vector Laboratories Inc., CA, USA), sealed and stored in the dark before taking fluorescence images. For rabbit wound sections, Masson staining was used to evaluate the wound status and angiogenesis. All samples were examined with an inverse confocal laser scanning microscope (TCS SL, Leica, Germany), and all images were analysed by ImageJ software. The mean number of lumens was calculated based on the average value of every 20x visual field.

1.10 Western blotting analysis

The cells were eluted from the hydrogels with trypsin. Cellular protein lysates were extracted from homogenized specimens in 150 mM NaCl, 10 mM Tris (pH 7.2), 0.1% sodium dodecyl sulfate (SDS), 1% Triton X-100, 1% deoxycholate, and 5 mM EDTA. The protein lysates were denatured in SDS-polyacrylamide gel electrophoresis (SDS-PAGE) loading buffer (Beyotime Biotechnology, China) at 100°C for 15 min. Then, the lysates were loaded on a 10% SDS-PAGE gel and subjected to electrophoresis under reducing conditions. Proteins were transferred onto a polyvinylidene difluoride membrane (Kodak, Rochester, NY) following the standard protocol. After blocking in 5% fat-free milk in PBS for 1 h, the membrane was incubated overnight with primary antibody at 4°C. The antibodies used in Western blotting were as follows: anti-HIF-1α (Affinity, AF-1009, 1:100) and anti-GAPDH (Millipore, MAB374, 1:5000). The samples were washed and then incubated with horseradish peroxidase-conjugated secondary antibody (Invitrogen, 1:4000) at RT. Detection was performed using enhanced chemiluminescence (ECL, GE Health, UK). The greyscale value of the stripe on the image was analyzed by ImageJ software. Repeat the experiment three times.

2. Results

The GelMA prepolymer was a white spongy object with a closed-pore lamellar structure on the surface. The cross-section showed a loose porous structure alternating vertically and horizontally. Physical images and field emission SEM (FE-SEM) images are shown in Supplementary Fig. 1A. The effect of the composition of the hydrogels on the ESR in PBS at a temperature of 4°C is illustrated in Supplementary Fig. 1B. The SW of the prepared composite hydrogels decreased with increasing β-cd content, and the water uptake showed the opposite trend.

The pH value of the prepolymer solution was always lower than that of the hydrogel, and decreased with the increase of β-CD content, while the hydrogel was on the contrary (Supplementary Table 1). When the hydrogels were immersed in different pH buffers at 37°C for 4 weeks, the quality of the hydrogels did not change significantly when the pH value was 7 but decreased when the pH value was 4 and increased obviously when the pH value was 10 (Supplementary Fig. 2A). Interestingly, in different concentrations of NaCl solution at 37°C for 4 weeks, the SW of the hydrogels increased with increasing β-cd content, which was opposite to that in PBS (Supplementary Fig. 2B). Overall, the hydrogels could remain relatively stable in an environment approximating the human ion concentration and pH value.

Degradation of hydrogels *In vitro* is shown in Supplementary Fig. 3A. The mass of all hydrogels first increased and then decreased, and the hydrogels basically degraded within 5 days. With the increase in the amount of β-cd, the degradation rate of the hydrogel increased *In vitro*. On the 5th day, a small amount of pure GelMA hydrogel remained, and the other groups had been completely degraded. As shown in Supplementary Fig. 3B, with the increase in implantation days, the volume of the subcutaneous residual hydrogels decreased, and the hydrogels were almost

completely degraded on the 21st day.

The ultrasonic elastography results showed that the hydrogels of all groups were soft, and the Young's modulus of the hydrogels increased with increasing amounts of β-cd (Supplementary Fig. 4A). A water CA test was used to detect the hydrophilicity of the hydrogels and showed that all groups of hydrogels exhibited hydrophilicity, which decreased with increasing β-cd addition (Supplementary Fig. 4B).

The key interface of the control module of integrated smart dressing was showed in Supplementary Fig. 5A and Supplementary Fig. 5B displayed the different working states of the microenvironment sensor In vitro, which included signal search, upon connection, display interface and turn off the switch. The assembly process of integrated smart dressing was shown in Supplementary Fig. 5C and the combination of hydrogel and biomimetic nanofibre membranes should not affect the monitoring performance of microenvironment sensors. Integrated smart dressings made of hydrogel and sensor chip could still realize real-time monitoring of wound microenvironment parameters (Supplementary Fig. 5D). The process of the application of integrated smart dressing on wound of full-thickness skin defect on the back of rabbit. The wound was established and fixed with a plastic ring, firstly. And nanofiber membrane was applied to the wound, then the hydrogel combined with the sensor was placed on the wound (Supplementary Fig. 5E).

Supplementary Fig. 6 showed the 3D shape of the lumen formed by planting HDMECs on the hydrogels. The three-dimensional lumen-like structures were formed in all groups, and the best morphology was obtained when the content of β-cd was 2% and 3%, which formed a vascular-like network.

The wound healing speed of diabetic rats was significantly slower than that of normal and

hydrogel-treated wounds. The wound healing speed of diabetic rats with hydrogel-treated wounds was close to that of normal wounds or even faster (Supplementary Fig. 7A). Immunohistochemical staining showed that only a small amount of abnormal angiogenesis could be seen in diabetic wounds. On the 7th day, a network of new small blood vessels was formed on normal wounds. The rate of new blood vessels was faster and the morphology was better after the application of hydrogel (Supplementary Fig. 7B).

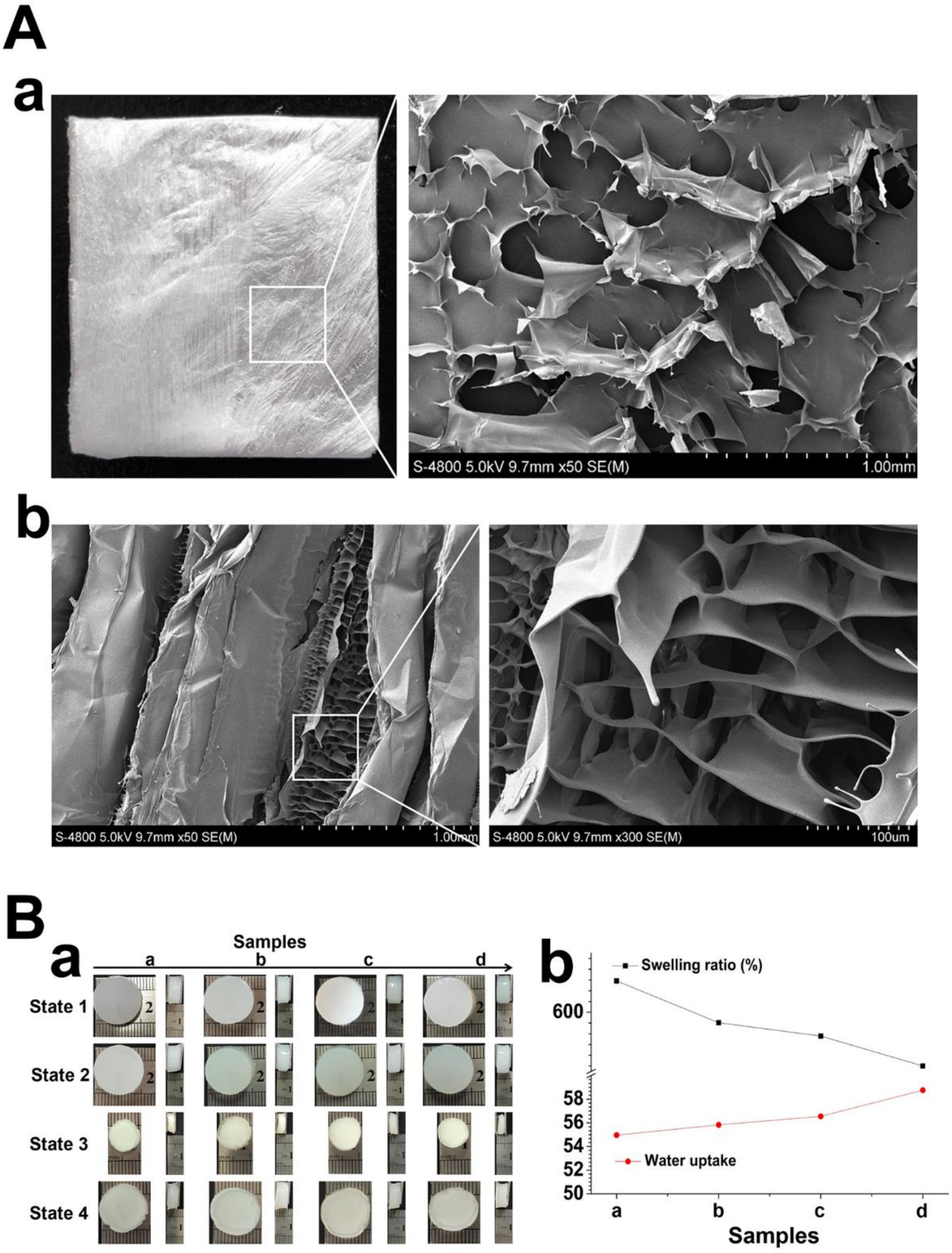

Supplementary figure 1. General photographs and ultrastructure of the GelMA prepolymer, swelling and water uptake of the GelMA+β-cd hydrogel. **A.** The GelMA prepolymer was a white spongy object with a closed-pore lamellar structure on the surface (a). The cross-section showed a loose porous structure alternating vertically and horizontally (b). **B.** a. Physical diagram of hydrogels in different states (State 1. Initial; State 2. Swelling; State 3. Dehydration; State 4.

Reswelling). b. Swelling ratio and water intake capacity of each hydrogel. The swelling ratio of the prepared composite hydrogels decreased with increasing β-cd content, and the water uptake showed the opposite trend. (Label descriptions: a. pure GelMA, b. with 2% β-cd, c. with 3% β-cd and d. with 4% β-cd.)

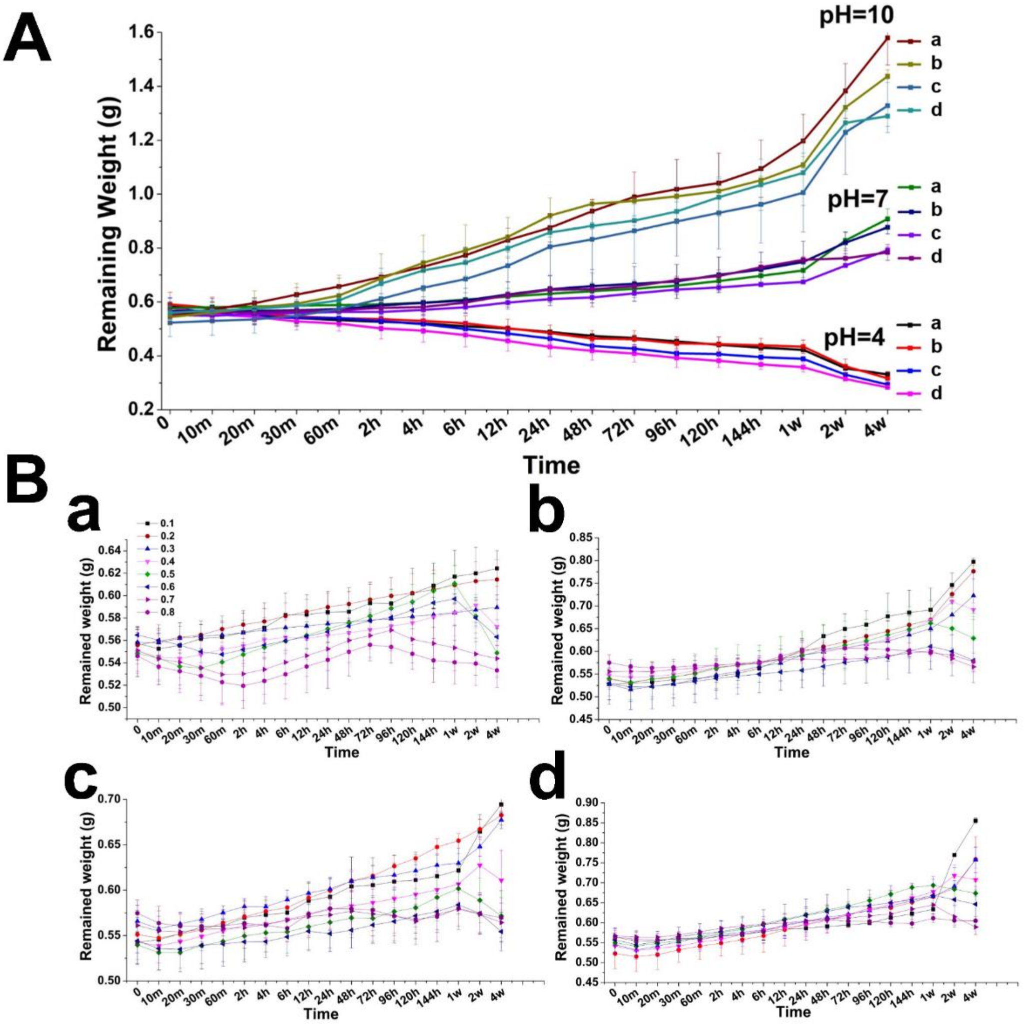

Supplementary figure 2. Swelling of hydrogels in solutions with different pH values or NaCl concentrations and *in vitro* degradation. **A.** Swelling of hydrogels in different pH solutions. When the hydrogels were immersed in different pH buffers at 37°C for 4 weeks, the weight of the hydrogels did not change significantly when the pH value was 7, but decreased when the pH value was 4, and increased significantly when the pH value was 10. **B.** Swelling of hydrogels in solutions

with different NaCl concentrations. At different concentrations of NaCl solution at 37°C for 4 weeks, the swelling ratio of the hydrogels increased with increasing β-cd content. (Label descriptions: a. pure GelMA, b. with 2% β-cd, c. with 3% β-cd and d. with 4% β-cd.)

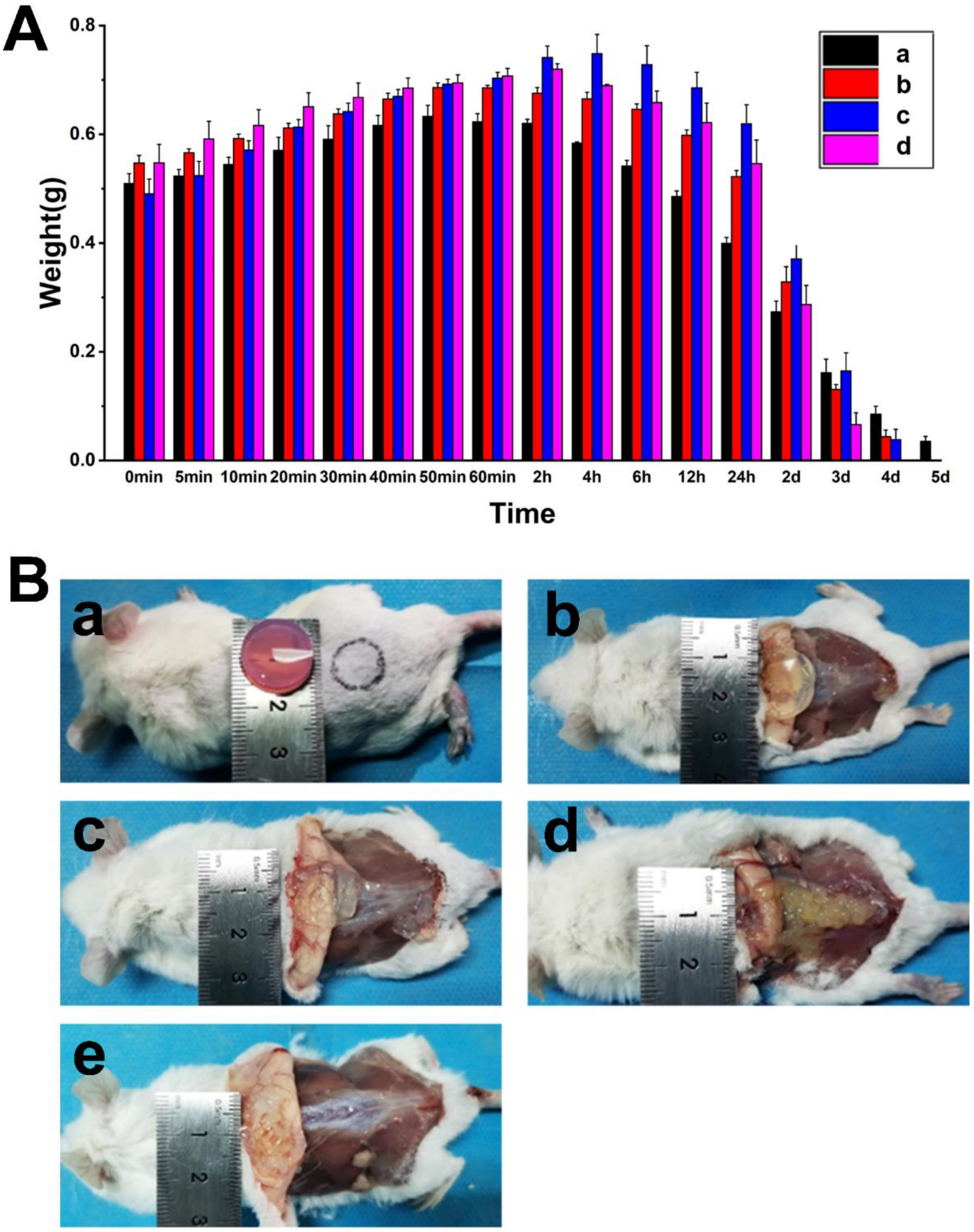

Supplementary figure 3. Degradation of hydrogels *in vitro* and *in vivo*. **A.** Degradation of hydrogels in 2 mg/mL type I collagenase solution. The mass of all hydrogels first increased and then decreased and basically degraded within 5 days. With the increase in the amount of β-cd, the

degradation rate of the hydrogel increased *in vitro*. On the 5th day, a small amount of pure GelMA hydrogel remained, and that in the other groups had been completely degraded. (Label descriptions: a. pure GelMA, b. with 2% β-cd, c. with 3% β-cd and d. with 4% β-cd.) **B.** Degradation of the hydrogel *in vivo*. With the increase in implantation days, the volume of subcutaneous residual hydrogels decreased, and they are almost completely degraded on the 21st day. a-e represent days 0, 3, 7, 14 and 21, respectively.

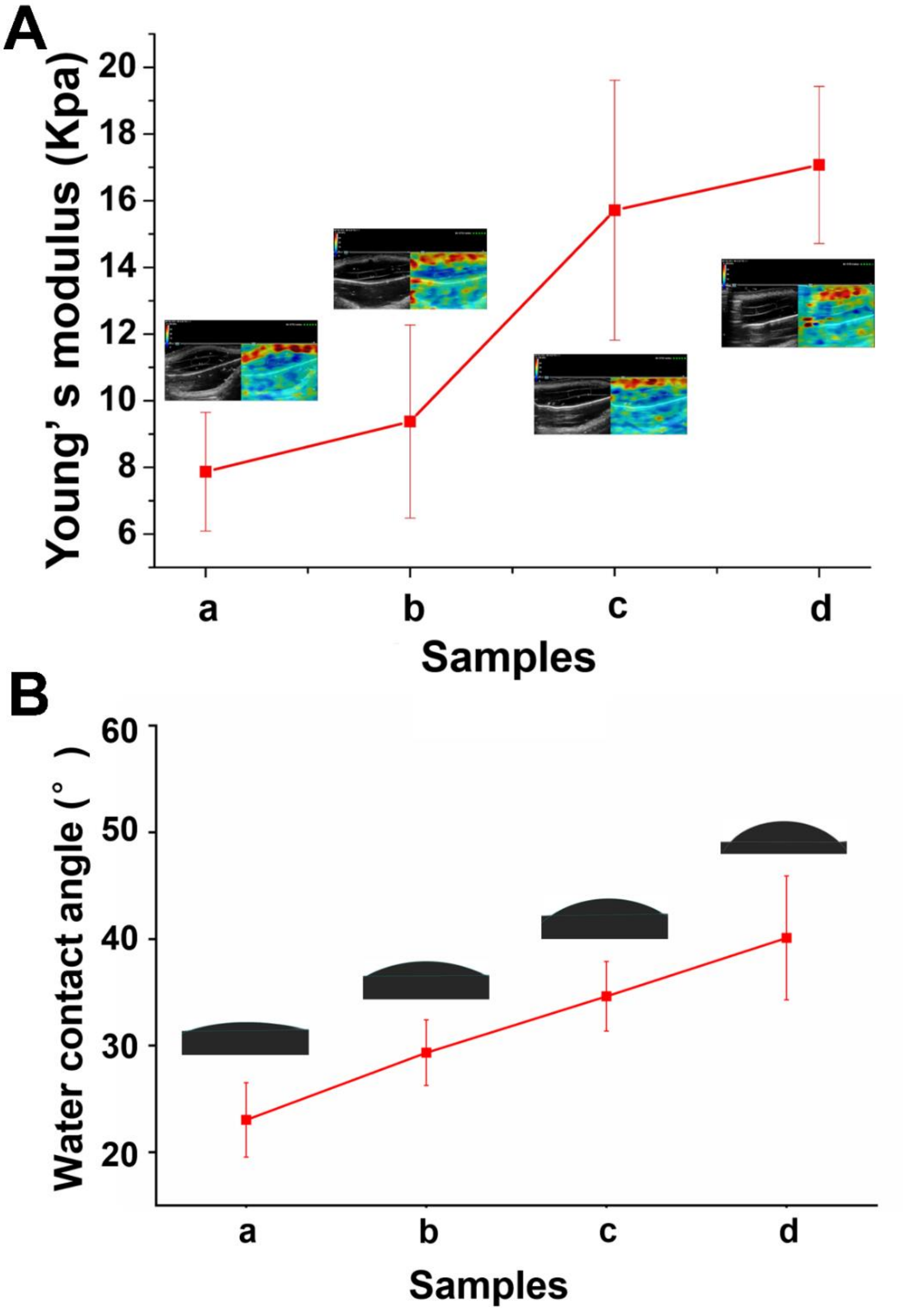

Supplementary figure 4. Shear wave elastography (SWE) and water contact angle (WCA) results of hydrogels. **A.** Young's modulus of hydrogels. The hydrogels in all groups were soft, and the Young's modulus of hydrogels increased with increasing β-cd content. **B.** Hydrophilicity of hydrogels. All groups of hydrogels were hydrophilic, and the hydrophilicity decreased with increasing β-cd content. (Label descriptions: a. pure GelMA, b. with 2% β-cd, c. with 3% β-cd and d. with 4% β-cd.)

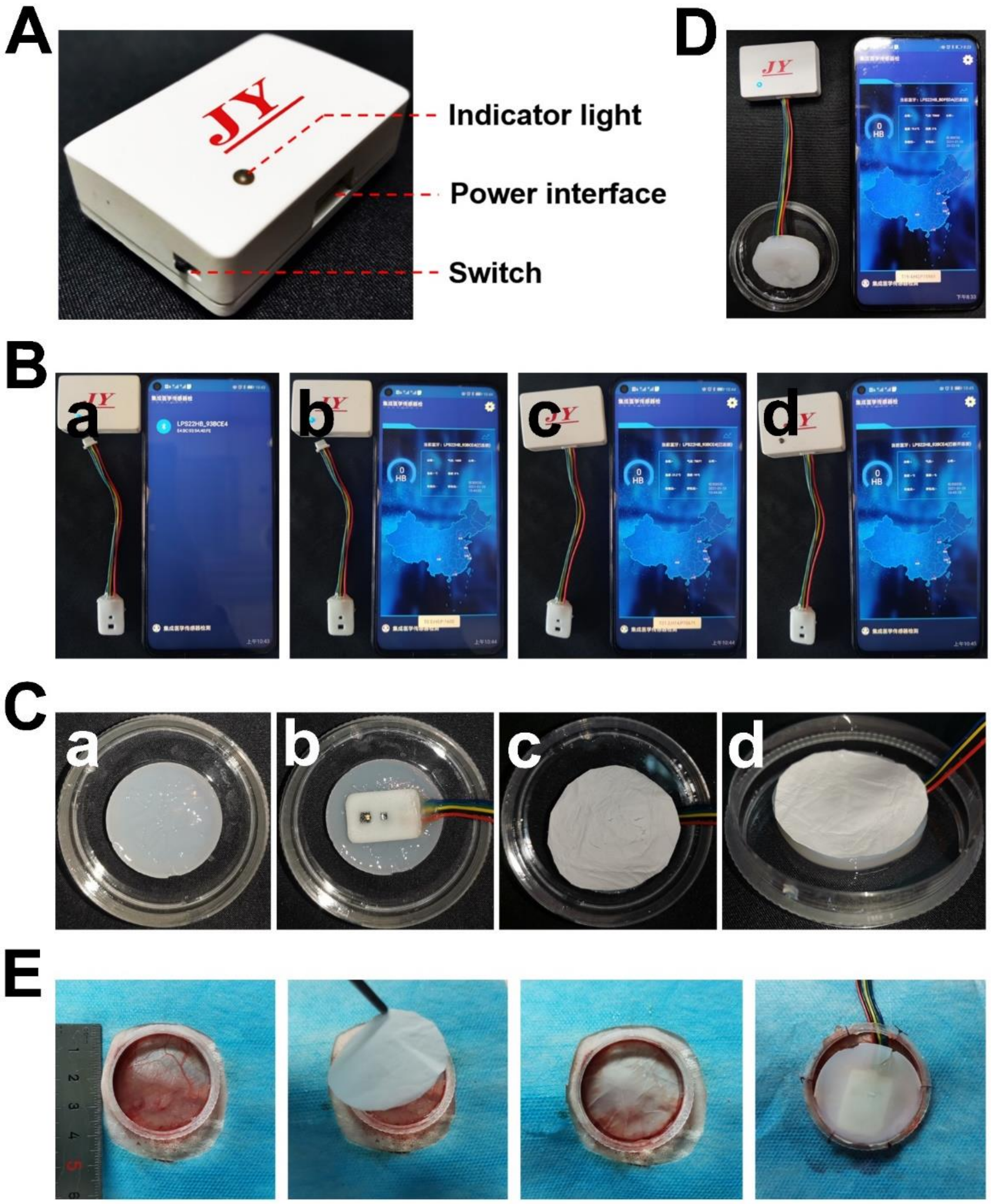

Supplementary figure 5. The key port of the control module, the different working states of the sensor and the construction process of the integrated smart dressing. **A.** The key interface of the control module of the integrated smart dressing. **B.** Different working states of the microenvironment sensor *in vitro*. a. Signal search. b. Upon connection. c. Display interface. d. Turn off the switch. **C.** The manufacturing process of the integrated smart dressing. **D.** Integrated smart dressings composed of hydrogel and sensor chips could still enable real-time monitoring of wound microenvironment parameters. **E.** The process of the application of integrated smart dressing on wounds of full-thickness skin defects on the back of rabbits. The wound was established and fixed with a plastic ring. A nanofibre membrane was applied to the wound, and the hydrogel combined with the sensor was then placed on the wound.

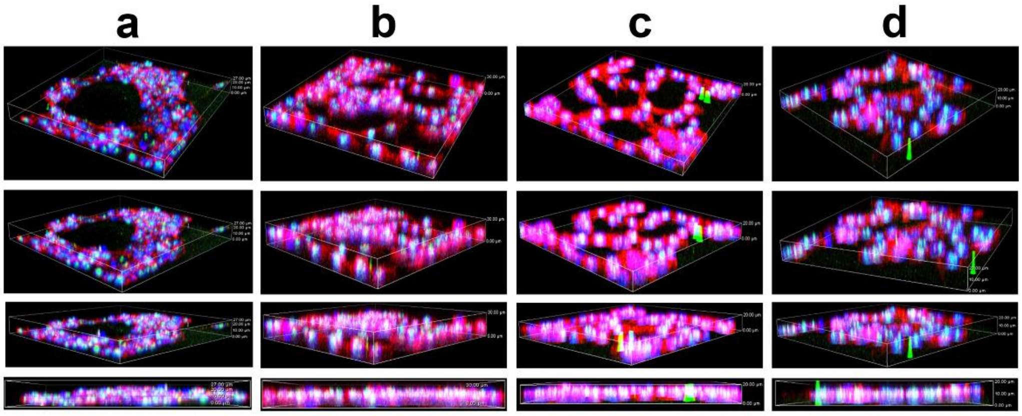

Supplementary figure 6. Three-dimensional image of lumen formed by HDMECs seeded on hydrogels. The three-dimensional lumen-like structures were formed in all groups, and the best morphology was in b and c group, which formed a vascular-like network. (Label descriptions: a. pure GelMA, b. with 2% β-cd, c. with 3% β-cd and d. with 4% β-cd.)

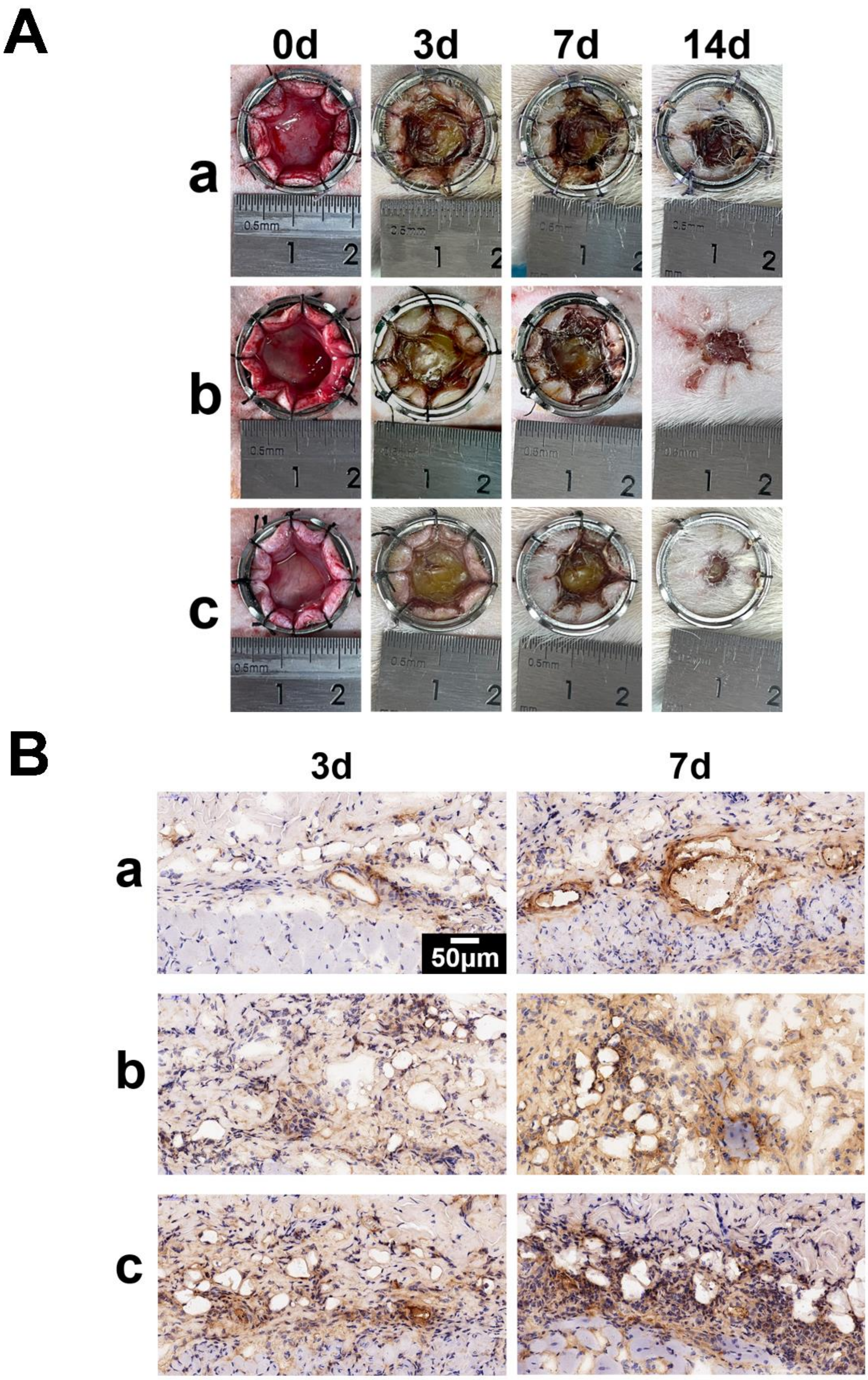

Supplementary figure 7. Application of hydrogels in chronic wounds. **A.** Images of the wound healing process of diabetic rats. The healing speed of normal or hydrogel-treated wounds was significantly faster than that of diabetic rat wounds, and the hydrogel-treated group was even the

fastest. **B.** Immunohistochemical images of the wounds of diabetic rats. Only a small amount of abnormal angiogenesis could be seen in diabetic wounds. On the 7th day, a network of new small blood vessels was formed on normal wounds. The rate of new blood vessels forming was faster and the morphology was better after the application of hydrogel. (Label descriptions: a. diabetic wound, b. normal wound and c. diabetic wound treated with the hydrogel.)

Supplementary Table 1. pH value of hydrogels before and after gelation with different β-cd amount (%).

β-cd amount (%)	Pre-Gel	Gel
0	5.53	5.62
2	5.51	5.66
3	5.50	5.69
4	5.50	5.71

Reference

Bulcke, A.I.V.D., Bogdanov, B., Rooze, N.D., Schacht, E.H., Cornelissen, M., and Berghmans, H. (2000). Structural and rheological properties of methacrylamide modified gelatin hydrogels. *Biomacromolecules* 1(1), 31-38.

Hoch, E., Schuh, C., Hirth, T., Tovar, G.E., and Borchers, K. (2012). Stiff gelatin hydrogels can be photo-chemically synthesized from low viscous gelatin solutions using molecularly functionalized gelatin with a high degree of methacrylation. *J Mater Sci Mater Med* 23(11), 2607-2617. doi: 10.1007/s10856-012-4731-2.

Reed, M.J., Meszaros, K., Entes, L.J., Claypool, M.D., Pinkett, J.G., Gadbois, T.M., et al. (2000). A new rat model of type 2 diabetes: the fat-fed, streptozotocin-treated rat. Metabolism 49(11), 1390-1394. doi: 10.1053/meta.2000.17721.
